# Multivariate GBLUP Improves Accuracy of Genomic Selection for Yield and Fruit Weight in Biparental Populations of *Vaccinium macrocarpon* Ait

**DOI:** 10.3389/fpls.2018.01310

**Published:** 2018-09-12

**Authors:** Giovanny Covarrubias-Pazaran, Brandon Schlautman, Luis Diaz-Garcia, Edward Grygleski, James Polashock, Jennifer Johnson-Cicalese, Nicholi Vorsa, Massimo Iorizzo, Juan Zalapa

**Affiliations:** ^1^Bayer CropScience NV, Innovation Center, Ghent, Belgium; ^2^The Land Institute, Salina, KS, United States; ^3^Department of Horticulture, University of Wisconsin Madison, Madison, WI, United States; ^4^Instituto Nacional de Investigaciones, Forestales, Agrícolas y Pecuarias, Campo Experimental Pabellón, Aguascalientes, Mexico; ^5^Valley Corporation, Tomah, WI, United States; ^6^Genetic Improvement of Fruits and Vegetables Laboratory, USDA-ARS, Chatsworth, NJ, United States; ^7^Blueberry and Cranberry Research and Extension Center, Rutgers University, Chatsworth, NJ, United States; ^8^Department of Horticulture Sciences, Plants for Human Health Institute, North Carolina State University, Kannapolis, NC, United States; ^9^Vegetable Crops Research Unit, USDA-ARS, University of Wisconsin, Madison, WI, United States

**Keywords:** genomic prediction, prediction accuracy, genomic selection, multivariate models, *Vaccinium macrocarpon*

## Abstract

The development of high-throughput genotyping has made genome-wide association (GWAS) and genomic selection (GS) applications possible for both model and non-model species. The exploitation of genome-assisted approaches could greatly benefit breeding efforts in American cranberry (*Vaccinium macrocarpon*) and other minor crops. Using biparental populations with different degrees of relatedness, we evaluated multiple GS methods for total yield (TY) and mean fruit weight (MFW). Specifically, we compared predictive ability (PA) differences between univariate and multivariate genomic best linear unbiased predictors (GBLUP and MGBLUP, respectively). We found that MGBLUP provided higher predictive ability (PA) than GBLUP, in scenarios with medium genetic correlation (8–17% increase with cor_g_~0.6) and high genetic correlations (25–156% with cor_g_~0.9), but found no increase when genetic correlation was low. In addition, we found that only a few hundred single nucleotide polymorphism (SNP) markers are needed to reach a plateau in PA for both traits in the biparental populations studied (in full linkage disequilibrium). We observed that higher resemblance among individuals in the training (TP) and validation (VP) populations provided greater PA. Although multivariate GS methods are available, genetic correlations and other factors need to be carefully considered when applying these methods for genetic improvement.

## Introduction

A central goal of genetics is the identification of genotype-phenotype associations. Traditional quantitative trait loci (QTL) mapping and genome-wide association studies (GWAS) are the primary tools for achieving such a goal. Thousands of genetic variants associated with traits of agronomic importance in economically important crops have been identified in the last century (Ingvarsson and Street, 2011). However, unraveling the causal genes behind such QTLs has often not been accomplished due to the high costs involved. Fortunately, the identification of markers in linkage disequilibrium (LD) with agriculturally important causal variants has been enough to move the genomic information to breeding applications such as marker-assisted selection (MAS), marker-assisted backcrossing, and pyramiding of major disease resistance genes (Flint-Garcia et al., 2003; Holland, 2004; Jiang et al., 2004; Bertrand and Mackill, 2008). However, after decades of studies, the application and value of the QTL paradigm for plant improvement has been questioned due to its low success in deploying genetic markers for breeding quantitative traits (Bertrand and Mackill, 2008; Xu and Crouch, 2008).

Genomic selection (GS), introduced by Meuwissen et al. (2001), has become the next step in MAS methods and has been effectively used in plant and animal breeding programs for more than a decade (Hayes et al., 2009; Jannink et al., 2010). Currently, several species have adopted this methodology, and moderate to high prediction accuracies [based on cross-validation (CV)] have been reported in crops such as wheat (*Triticum aestivum*), oat (*Avena sativa*), maize (*Zea mays*), rice (*Oryza sativa*), rye (*Secale cereale*), and barley (*Hordeum vulgare*) (Asoro et al., 2011; Zhao et al., 2012; Lipka et al., 2014; Rutkoski et al., 2014; Wang et al., 2014; Sallam et al., 2015; Spindel et al., 2015). Fruit crops have adopted this technology slower, although major fruit crops such as apple (*Malus* × *domestica*) and kiwifruit (*Actinidia deliciosa*) have made great progress on the implementation of these technologies (Testolin, 2010; Kumar et al., 2012; Muranty et al., 2015). The slower adoption could be due to the availability of genomic resources, and concerns about the effectiveness of GS compared to classical methods, such as phenotypic recurrent selection, which have made important progress in fruit breeding for hundreds of years. Recently, next-generation sequencing (NGS) studies have reduced the gap between major and minor crops such as cranberry (*Vaccinium macrocarpon* Ait.; 2n = 2x) (Huang et al., 2009; Zalapa et al., 2012; Fajardo et al., 2014; Polashock et al., 2014; Schlautman et al., 2015; Covarrubias-Pazaran et al., 2016). Other fruit crops, including apple and kiwifruit, have used these methods to generate vast quantities of markers to propose and perform GS (Testolin, 2010; Kumar et al., 2012; Muranty et al., 2015). The efficiency of GS to select parents in shorter intervals (i.e., predictions early on the breeding pipeline) and the possibility to increase selection intensity compared to classical approaches (i.e., ability to predict untested individuals) holds great potential for fruit breeding (Riedelsheimer and Melchinger, 2013; Endelman et al., 2014).

Various factors including training population (TP) size, marker density, heritability, magnitude of the LD, trait architecture, resemblance between TP and the validation population (VP), and the interaction of these factors, appear to be the principal forces driving the prediction accuracies of GS (Lorenzana and Bernardo, 2009; Guo et al., 2012; Resende et al., 2012; Habier et al., 2013; Riedelsheimer et al., 2013; Lorenz and Smith, 2015; Muranty et al., 2015). In addition, a thorough characterization and modeling of environmental variances (Technow et al., 2015) and the covariance among multiple traits also appear to increase the accuracy of GS models.

One of the most recent ideas to increase the predictive ability of the GS models is the use of multivariate models. The use of multivariate mixed models in breeding was originally proposed in animal breeding to model the genetic correlation among traits, longitudinal data, and to model genotype by environment interactions (trajectory across multiple years or environments) in order to exploit the existent correlations in the data (Mrode, 2014; Lee and Van der Werf, 2016). The first application of mixed models for multi-trait evaluation was by Henderson and Quaas (1976). The gain in accuracy of multivariate models compared to univariate models depends largely on the difference between the genetic and residual correlations between the responses (Schaeffer, 1984; Thompson and Meyer, 1986). A positive impact of the multi-trait methodology is its capacity to increase the predictive ability on traits with low heritability when they are analyzed together with high heritability traits that are genetically correlated (Thompson and Meyer, 1986). Until the last decade, multivariate methods have been exploited in plant and animal breeding mainly in species with pedigree information available to model the relationships among individuals and traits in the mixed model framework (Mrode, 2014). With the advent of massive molecular marker datasets, genomic relationship matrices are replacing pedigree-based relationship matrices, opening new analysis options for crops with limited pedigree information (Endelman and Jannink, 2012).

Like other woody perennial species, cranberry genetic improvement has been limited by the long interval needed to produce a cultivar (Janick and Moore, 1975; Johnson-Cicalese et al., 2015). Furthermore, due to its recent domestication in the mid-1800s and late start of breeding efforts in the 1920s, advances in cranberry genetics have been even slower with respect to other major fruit crops such as apple and peach. Therefore, cranberry could serve as a model for how NGS coupled with molecular-assisted breeding strategies, such as GS, could accelerate cultivar development in non-model or partially domesticated crop species (Zalapa et al., 2012, 2015). Within the past 5 years, NGS technologies have been used to increase the availability of genomic resources in cranberry from almost none to now include: assembled organellar genomes (Fajardo et al., 2012, 2014), a draft nuclear genome and transcriptome (Polashock et al., 2014), multiple SSR based genetic maps (Georgi et al., 2013; Schlautman et al., 2015), and most recently high density genetic maps and a consensus map with thousands of SNP (Covarrubias-Pazaran et al., 2016; Schlautman et al., 2017) and the use of massive high throughput phenotyping techniques (Diaz-Garcia et al., 2018). Currently, cranberry breeding relies heavily in the evaluation of medium to large biparental populations with the main goal of improving commercially useful traits such as fruit color, shape, and brix degrees, as well as disease resistance and yield. Cranberry breeding requires a hefty initial economic investment for field evaluation due to the need of constructing flooding beds that mimic commercial growing conditions to allow water harvesting. Construction of a one acre cranberry bed to evaluate 500 genotypes will cost between $25,000 and $30,000 USD, not including maintenance and evaluation of the bed. Additionally, the release of a new cranberry varieties has required more 20 years on average. Thus, reducing the breeding cycle length by using genomic technologies and selective phenotyping to reduce the high cost of evaluating biparental populations are the main drivers of current research in cranberry breeding.

In this research, we used the genomic resources available in cranberry to test the usefulness of genomic selection and compare differences in PA for total yield (TY) and mean fruit weight (MFW) in cranberry. We used both univariate and multivariate genomic best linear unbiased predictor (GBLUP and MGBLUP, respectively) approaches together with traditional biparental populations commonly used in cranberry breeding. This research will allow us to understand the benefits of using genomic prediction using related individuals (i.e., full-sib and half-sib individuals) with the aim of reducing the population-sizes of families to be planted for field evaluation (which is the most expensive part of a cranberry breeding program) while also increasing the number of families evaluated in the field trials. Also, we investigated two scenarios: low or null genetic correlation scenario (in our data the correlation between TY and MFW) and high genetic correlation scenario (in our data the correlation among multiple years). These two scenarios will allow us to investigate the usefulness of MGBLUP to improve the PA in our current GS efforts.

## Materials and methods

### Plant material and marker information

We used three cranberry biparental populations denominated CNJ02 (Mullica Queen x Crimson Queen; MQ × CQ, *N* = 148), CNJ04 (MQ × Stevens, *N* = 67) and GRYG [BGBLNL × (GH1x35), *N* = 351]. The parents of the three crosses are highly heterozygous genotypes frequently used in cranberry breeding programs. MQ and CQ are hybrids obtained after three generations of selection from wild materials, BGBLNL and GH1x35 are second-generation hybrids and Stevens is a first-generation hybrid from two wild selected parents. The CNJ02 and CNJ04 populations are planted and maintained at the Rutgers University P.E. Marucci Center, Chatsworth, NJ. The GRYG population is planted and maintained at Valley Corporation, Tomah, Wisconsin. CNJ02 and CNJ04 are half-sibs, and are not closely related with GRYG. Each genotype was clonally propagated and planted in the field using multiple cuttings in a defined 0.46 m^2^ (5 ft^2^) square plot to mimic commercial conditions.

Genotypic information was obtained using the GBS protocol from Elshire et al. (2011) with modifications described in Schlautman et al. (2017). EcoT22I, which cuts the site 5′-ATGCA↓T-3′/ /3′-T↑ACGTA-5′, was selected for reducing genome complexity in this study based on GBS optimization results in cranberry to ensure good coverage for sequence tags in all populations [more details can be found in Covarrubias-Pazaran et al. (2016) and Schlautman et al. (2017)]. Resulting libraries were sequenced on the Illumina HiSeq 2000 sequencing platform (Illumina, San Diego, California).

From the different number of SNPs available in each of the three biparental populations, a total number of 7389 SNP markers were polymorphic across the 12 linkage groups (LGs) in at least one of the three cranberry populations. Markers were positioned using the consensus genetic map (anchoring 6074 markers) obtained and described by Schlautman et al. (2017). Only biallelic loci with minor allele frequency (MAF) >0.05 were used in the analyses. According to the genetic maps published the SNPs cover the entire linkage groups and therefore causal and non-causal regions were assumed to have markers. Genotypic data is available in the Supplementary File 1.

### Phenotype collection

Repeated measures for total yield (TY) and mean fruit weight (MFW) were taken over a three-year period for 148 genotypes from the CNJ02 population (2011–2013) and 67 genotypes for CNJ04 (2012-2014); the GRYG population comprised 351 genotypes for which data was collected over a two-year period (2014-2015). TY was determined by harvesting and weighting all the fruit within a 0.09 m^2^ (1 ft^2^) metallic square set in each cranberry plot [0.46 m^2^ (5 ft^2^)] representing each genotype. Twenty five fruit for each genotype were randomly selected and weighted to calculate MFW as described in Georgi et al. (2013) and Johnson-Cicalese et al. (2015).

### Experimental design and mixed modeling

All populations were planted together with 15 check plots (3 plots per 5 parents) positioned spatially across the flooding beds (commercial-condition fields). Additionally, to deal with the lack of replication in our experimental design, a two-step approach was used for the GS exercise for each population. First, a heterogeneous-variance univariate mixed model including all years of data was used to fit a model of the form y = **X**β + **Z**u + ε, where y was the response variable (TY or MFW), **X** and **Z** were incidence matrices for fixed and random effects respectively, β was the vector of fixed effects associated to the environment (year-location combination), u was the vector of random effects associated to rows [r ~ (0, **I**$\sigma_{\text{r}}^{2}$)], range or columns [c ~ (0, **I**$\sigma_{\text{c}}^{2}$)], the 2-dimensional spline [d ~ (0, **I**$\sigma_{\text{d}}^{2}$)], and genotypic effects [g~ (0, **I**$\sigma_{\text{g}}^{2}$)] (no marker information used at this point), and ε was the error associated to the model ε ~ (0, **I**$\sigma_{\text{e}}^{2}$). The heterogeneous variance model was used to allow a different variance component for genotype effects in each environment as for the other random effects. This was achieved by using the *diag()* covariance structure functionality in the *mmer2()* function available in *sommer*, i.e., *diag(ENV):genotype* fits for a random effect for genotypes with a variance var(u_g_) = G_e_ ⊗ A, where G is the variance covariance for genotypes among environments and A is a relationship matrix among genotypes:

$$\begin{array}{l} \text{var}(u_{g})=G_{e}⊗A=\left[\begin{array}{ccc} \sigma_{e1}^{2} & \ldots & \sigma_{e1ei}\\ \ldots & \ddots & \ldots \\ \sigma_{eie1} & \ldots & \sigma_{eiei}^{2} \end{array}\right]⊗A \end{array}$$

where A was typically a variance covariance matrix among the levels of the random effect (i.e., genotypes evaluated, levels of blocks, etc.) and for this model was a diagonal matrix with as many ones as genotypes evaluated (the genomic relationship matrix was not used at this point) and σ_eiej_ was the covariance among the same genotypes in different environment and here was considered zero for the diagonal model. The result was that different variance components can be estimated for each random effect in each environment, and by-environment genotype predictions can be obtained. Because the mapping populations were full-sib families with replication of alleles across genotypes in a uniformly managed cranberry bed, we made a spatial relationship assumption stating that large rows and columns of genotypes should resemble one another allowing to fit row and column effects (Schlautman et al., 2015). In addition, we fitted the two-dimensional splines to account for spatial trends that reflect shapes proper of tensor products (Velazco et al., 2017). Residuals were investigated using variograms to verify the proper fit. All spatial mixed models (two-dimensional splines) were fitted using the R package *sommer* (Covarrubias-Pazaran, 2016). Variance components were tested to be different from zero using likelihood ratio tests. Description of the phenotypic data, variance components and heritabilities for this first step modeling can be found in the Additional File 1.

From these models we obtained two types of predictions for the genotype effect, one across environments and another for each environment. The idea was to use the by-environment genotype prediction to fit a multivariate model using each environment genotype predictions as a response from the same trait (i.e., [y_MFW−2011_, y_MFW−2012_]) to mimic a natural high genetic correlation scenario, whereas the across-environment predictions for both traits were used to build the multivariate response that in our data mimics a low genetic correlation scenario given the low genetic correlation found among these traits (i.e., [y_MFW_, y_TY_]).

### Data filtering

In our experience, the use of data from environments with null or very small genomic-heritability values (i.e., ${\text{h}}_{\text{g}}^{2}$ < 0.10) in multivariate models tends to bring computational issues or non-sense genetic correlation values. Therefore, we decided to calculate genomic heritabilities for each environment using the by-environment genotype prediction as response and a single random effect for genotypes using the genomic relationship matrix. In summary a model of the form y = **X**β + **Z**u + ε, where y is the response variable (by-environment genotype prediction for TY or MFW), **X** and **Z** are incidence matrices for fixed and random effects respectively, β is the vector of fixed effects associated to the intercept only, u is the vector of random effects associated to genotypes [g ~ (0, **A**$\sigma_{\text{r}}^{2}$)], where A is the additive genomic relationship matrix [**A**_g_ = **MM**'/2 Σ p_i_(1-p_i_)] (VanRaden, 2008). Genomic heritabilities instead of generalized forms of heritability where calculated given the greater ability of genomic heritability to provide insight on the PA of the data (Cullis et al., 2006; de los Campos et al., 2015). For each trait-year combination the genomic heritability was calculated using the formula ${\text{h}}_{\text{g}}^{2}$ = $\sigma_{\text{g}}^{2}$ / ($\sigma_{\text{g}}^{2}$ + $\sigma_{\text{e}}^{2}$), where $\sigma_{\text{g}}^{2}$ is the genetic variance using marker-based relationship and $\sigma_{\text{e}}^{2}$ is the residual variance. Standard errors for the heritabilities were computed using the delta method implemented in the pin function of the R package *sommer* (Covarrubias-Pazaran, 2016). Environments (year-location combination) with ${\text{h}}_{\text{g}}^{2}$ lower than 0.10 or with SE that approximated the ${\text{h}}_{\text{g}}^{2}$ to zero were discarded from all posterior analyses.

### Genetic correlation across years

Multivariate mixed models were used to assess the genetic correlation across years within populations. Following (Maier et al., 2015), the multivariate mixed model implemented has the form:

$$\begin{array}{c} {\text{y}}_{1}\text{ = }X_{\text{1}}\beta_{\text{1}}\text{ + }Z_{\text{1}}{\text{u}}_{1}\text{+ }{\text{e}}_{1}\;\\ {\text{y}}_{2}\text{ = }X_{\text{2}}\beta_{\text{2}}\text{ + }Z_{\text{2}}{\text{u}}_{2}\text{+ }{\text{e}}_{2}\;\\ \vdots \\ {\text{y}}_{t}\text{ = }X_{t}\beta_{t}\text{ + }Z_{t}{\text{u}}_{t}\text{ + }{\text{e}}_{t} \end{array}$$

where y_i_ is a vector of trait phenotypes, β_i_ is a vector of fixed effects, u_i_ is a vector of random effects for individuals and e_i_ are residuals for trait “I” (i = 1, …, t). The random effects (u_1_ … u_i_ and e_i_) are assumed to be normally distributed with mean zero. **X** and **Z** are incidence matrices for fixed and random effects respectively. The distribution of the multivariate response and the phenotypic variance covariance (**V**) are:

$$\begin{array}{l} \text{Y=}X\beta \text{+}Z\text{u+}\varepsilon \text{ where Y}\sim \text{MVN}(X\beta ,V)\\ y=\left[\begin{array}{c} y_{1}\\ \vdots \\ y_{t} \end{array}\right]X=\left[\begin{array}{c} X_{1}\;\ldots \;0\\ \ldots \;\ddots \;\ldots \;\\ 0\;\ldots \;X_{t} \end{array}\right]\\ V\;=\;\left[\begin{array}{ccc} Z_{1}K\sigma_{u1_{t1}}^{2}Z_{1}{}^{\prime }+Z_{1}R\sigma_{e_{t1}}^{2}Z_{1}{}^{\prime } & \ldots & Z_{1}K\sigma_{u1_{1,t}}Z_{t}{}^{\prime }+\;Z_{t1}R\sigma_{e_{1,t}}Z_{ti}{}^{\prime }\\ \vdots & \ddots \; & \vdots \\ Z_{1}K\sigma_{u1_{1,t}}Z_{t}{}^{\prime }+\;Z_{1}R\sigma_{e_{1,t}}Z_{t}{}^{\prime } & \ldots & Z_{t}K\sigma_{u1_{t}}^{2}Z_{ti}{}^{\prime }+\;Z_{t}R\sigma_{e_{t}}^{2}Z_{t}{}^{\prime } \end{array}\right]\; \end{array}$$

where **K** is the relationship or covariance matrix for the kth random effect (*u* = 1,…,k), and **R** = **I** is an identity matrix for the residual term. The terms, $\sigma_{uk_{i}}^{2}$ and $\sigma_{e_{i}}^{2}$ denote the genetic (or any of the kth random terms) and residual variance of trait “i,” respectively and σ_*u*_*k*__*ij*__and σ_*e*_*ij*__ the genetic (or any of the kth random terms) and residual covariance between traits “i” and “j” (*i* = 1,…,t, and *j* = 1,…,t). For more details about the multivariate algorithm used in *sommer* please look at Covarrubias-Pazaran (2018). The genetic correlation among years was calculated using the by-environment genotype predictions as the multivariate response.

### Model comparison

By-environment and across genotype predictions were used for validating univariate and multivariate GS in each population independently. The following methods were compared: (1) genomic best linear unbiased predictor (GBLUP), which used the information from all markers coded in the additive relationship matrix, (2) GBLUP-AD, which included the additive and dominance relationships, (3) GBLUP-ADE, which included the additive, dominance, and epistatic relationships, and (4) Multivariate GBLUP, which exploits the covariance information among traits (or environments) at the level of genotypes and residuals. These models were fitted using the *sommer* package (Covarrubias-Pazaran, 2016).

The first comparison among all models was made environment by environment and trait by trait (i.e., comparison among models for MFW in environment Y2011, Y2012, etc.) for each population using the by-environment genotype predictions as response variable. The MGBLUP for this first comparison used as the multivariate response the same trait-environment response than the univariate models plus data of an additional environment (high genetic correlation in our data). A second comparison among models was made using across-environment genotype predictions for each trait. The MGBLUP for this second comparison used as the multivariate response the across-environment genotype predictions for both traits (low genetic correlation scenario in our data).

The models were fitted using all markers by creating the additive genomic relationship matrix **A**_g_ for prediction in a kinship-based model [**A**_g_ = **MM**'/2 Σ p_i_(1-p_i_)] (VanRaden, 2008), dominance relationship matrix **D**_g_ [**D**_g_ = **NN**'/Σ 2p_i_q_i_(1- p_i_q_i_)] (Su et al., 2012) and additive by additive epistatic relationship matrix **E**_g_ (**E**_g_ = A#A; where # is the Hadamard product) (Su et al., 2012), where **M** is the marker matrix coded as −1, 0, 1 for the number of reference alleles for a given biallelic marker for the A matrix computation and 0, 1 (0 for homozygotes and 1 for heterozygotes genotypes) for the D matrix computation. The model used has the typical mixed model form; y = **X**β + **Z**u + ε, where y is the response variable, **X** and **Z** are incidence matrices for fixed and random effects, respectively, β is the vector of fixed effects (intercept only), u is the vector of random effects associated to the genotypic effects with the corresponding relationship matrices. For the multivariate GBLUP model only the additive relationship matrix was used, and the model and distributions follow Covarrubias-Pazaran (2018). In total, 100 iterations of 5-fold CV were used to test the PA under the different models. Tables and figures comparing the different models were built using the R Core Team (2017).

### Effect of marker density in prediction

To examine the influence of the number of markers in the PA, we fitted the univariate GBLUP model constructing the genomic relationship matrix (**A**_g_) with different number of markers equally spaced and covering the entire genome across the 12 LGs in cranberry (Lorenzana and Bernardo, 2009). The consensus map developed by Schlautman et al. (2017) was used to ensure a homogeneous marker distribution. Then, we divided the entire linkage distance (~1,250 cM) in different number of bins; 20, 50, 100, 250, 500, 750, 1,000 and bins to reach the following marker densities; 1 marker every 60, 24, 12, 4.8, 4.4, 1.6, and 1.2 cM. For example, in the first case we built the A matrix with 20 markers, one marker every 60 cM, and in the densest case with 1,000 markers, picking one marker at about every 1.2 cM. The PA was deduced for both TY and MFW by averaging the results from 100 iterations of 5-fold CV for both traits where the 5-fold strategy consisted in dividing the population in 5 groups and using 1 group as VP and the rest as TP (100 rounds of this strategy yields 500 data points). Results were recorded and plotted using R (R Core Team, 2015). This analysis was performed using across-environment genotype predictions for both traits.

### Effect of training population relationship in prediction

Following Lorenz and Smith (2015) the effect of resemblance between the TP and VP on the PA was examined in the three biparental populations. The three populations were chosen based on their degree of relationship. CNJ02 (MQ × CQ) were half-sibs with CNJ04 (MQ × Stevens). The GRYG population (BGBLNL95 × [GH1x35]) had little relationship with CNJ02 and CNJ04. Using the across-environment genotype predictions we fixed each population as the VP and the resemblance of the TP was varied using individuals with no relationship to the VP, related half-sib individuals (when available), and related full-sib individuals (within population). In total, 100 iterations of 5-fold CV were used to test the PA under the different scenarios.

### Data availability

Supplementary File 1 (SF1) contains the phenotypic and genotypic data. The R script for the analysis can be found in the Supplementary Files 2–5.

## Results

### Genomic heritabilities

After the initial spatial modeling, we used the by-environment genotype predictions to calculate the genomic heritability for each environment and trait combination. We found higher genomic heritabilities for MFW compared to TY. For example for GRYG's population, we found a genomic heritability of 0.22 for TY in 2014 whereas the same year gave a genomic heritability for MFW of 0.43 (Table 1). The same trend was found in the three populations across most years. Some years resulted in a very low genomic heritability (< 0.10 and close to zero using the SE of the ${\text{h}}_{\text{g}}^{2}$). Such years of data were removed from posterior analysis due to our experience that using genotype predictions with null or close to zero genomic heritability provides spurious predictions or non-sense estimates of genetic correlation when used in the multivariate framework. The heritability was higher in GRYG than in CNJ02, and the smallest in CNJ04. Removing the year-trait combinations with low heritability for posterior analysis resulted in 2 years of data for GRYG and CNJ02, and 1 year of data for CNJ04 for both traits TY and MFW.

**Table 1:** Year-base genomic heritabilities (h^2^g estimate) and their standard error (h^2^g SE) for three biparental populations (CNJ02, *N* = 148; CNJ04, *N* = 67; GRYG, *N* = 351) for traits total yield (TY) and mean fruit weight (MFW).

**Population**	**Year**	**Trait**	Removed^*^	**${\text{h}}_{\text{g}}^{2}$ estimate**	**${\text{h}}_{\text{g}}^{2}$ SE**
GRYG	Y2014	TY	No	0.228	0.080
GRYG	Y2015	TY	No	0.332	0.085
CNJ02	Y2011	TY	No	0.163	0.127
CNJ02	Y2012	TY	No	0.184	0.128
CNJ02	Y2013	TY	Yes	0.097	0.133
CNJ04	Y2011	TY	Yes	0.092	0.258
CNJ04	Y2012	TY	No	0.204	0.261
CNJ04	Y2014	TY	Yes	0.018	0.252
GRYG	Y2014	MFW	No	0.436	0.084
GRYG	Y2015	MFW	No	0.400	0.086
CNJ02	Y2011	MFW	No	0.562	0.118
CNJ02	Y2012	MFW	No	0.307	0.132
CNJ02	Y2013	MFW	Yes	0.059	0.115
CNJ04	Y2011	MFW	Yes	0.092	0.258
CNJ04	Y2012	MFW	No	0.204	0.261
CNJ04	Y2014	MFW	Yes	0.018	0.252

* *Posterior analysis based on multivariate mixed models were not calculated when the genomic heritability for the univariate models was < 0.10*.

### Genetic correlations

Given that repeated measures of TY and MFW were taken for the three biparental populations across different years (environments) in the 2011–2015 interval, genetic correlations between years within traits, and genetic correlation between traits were obtained using multivariate mixed models (Table 2). We found a high genetic correlation between years for the trait MFW in both GRYG and CNJ02 populations (i.e., 0.93), which indicates a good consistency of breeding values (BV) across years (Table 2). Additionally, the genetic correlations between years for TY for both populations were smaller compared to MFW, but still relatively high (i.e., 0.62–0.90; Table 2). On the other hand, the genetic correlation between TY and MFW using across-environment genotype predictions were close to zero. The standard error of the genetic correlations indicates that for GRYG and CNJ02 the genetic correlations are not different than zero, whereas for CNJ04 the genetic correlation was different than zero but with a very high SE due to the population size (*N* = 67).

**Table 2:** Genetic correlation between years within traits, among traits (rg estimate), and their standard errors (h^2^g SE) in three biparental populations (CNJ02, *N* = 148; CNJ04, *N* = 67; GRYG, *N* = 351).

Population^*^	**Cor type**	**r_g_ estimate**	**r_g_ SE**
GRYG	TY-MFW	−0.010	0.209
CNJ02	TY-MFW	−0.297	0.386
CNJ04	TY-MFW	0.880	0.412
GRYG	TY2014-TY2015	0.629	0.191
CNJ02	TY2011-TY2012	0.905	0.259
GRYG	MFW2014-MFW2015	0.931	0.080
CNJ02	MFW2011-MFW2012	0.934	0.107

* *Population CNJ04 had only 1 year of data left after filtering data based on genomic heritability making the calculation of MGBLUP for years impossible*.

### Model comparison

**Figure 1:**
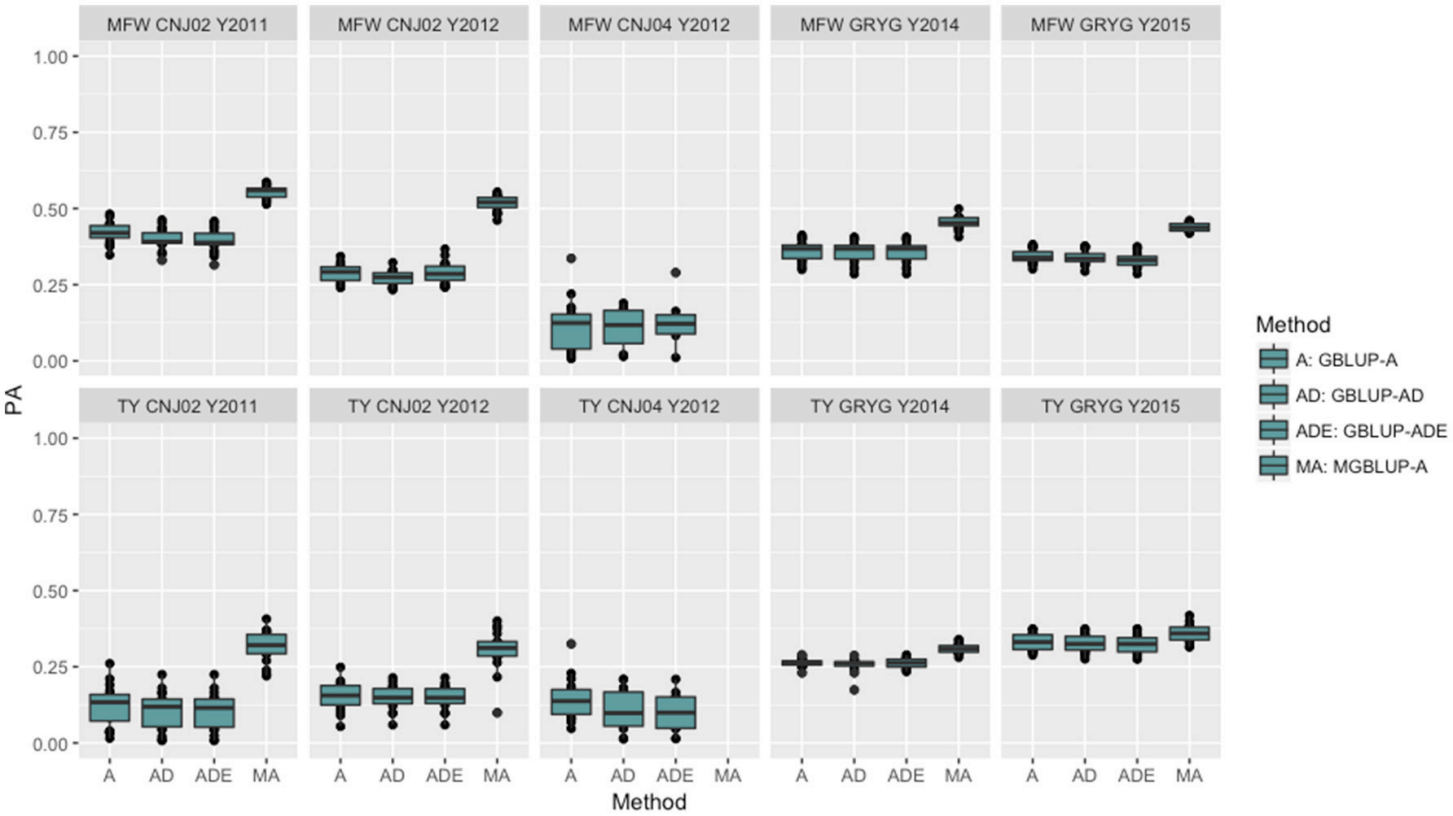
Year-based comparison between univariate and multivariate genomic best linear unbiased prediction methods (GBLUP and MGBLUP) for mean fruit weight (MFW) and total yield (TY) in three cranberry biparental populations. Methods within boxplots are GBLUP using only additive relationship matrix (GBLUP-A), GBLUP using additive and dominance relationship matrices (GBLUP-AD), GBLUP using additive, dominance and epistatic relationship matrices (GBLUP-ADE), and multivariate GBLUP using only additive relationship matrix (MGBLUP). MGBLUP used an additional year of data to form the multivariate response and the genetic correlation among these responses (high genetic correlation scenario).

The four genomic prediction methods compared; GBLUP-A, GBLUP-AD, GBLUP-ADE, and MGBLUP were performed by population to reflect two difference scenarios, the effect in PA using multivariate models under high and low genetic correlation. For the high genetic correlation scenario we used by-environment genotype predictions as a response (where MGBLUP uses as multivariate response the predictions for two environments with high genetic correlation). For TY, after 100 iterations of CV (complete sets of 5-fold), we found MGBLUP to be superior compared to GBLUP-A, GBLUP-AD, GBLUP-ADE in all environments and populations, except for CNJ04 where only 1 year of data was available and MGBLUP was not possible to evaluate (Figure 1; Table 2). For example, we found an increase in PA from r_TY−GBLUP_ = 0.12 to r_TY−MGBLUP_ = 0.32 in the CNJ02 population in the year 2011 when using GBLUP versus MGBLUP which used an additional year of data as an additional response (which had a genetic correlation of 0.90 ± 0.25). Similarly, in the same population in 2012 we found an increase of PA from r_TY−GBLUP_ = 0.15 to r_TY−MGBLUP_ = 0.30 between GBLUP in MGBLUP. In the GRYG population in 2014 we found an increase from r_TY−GBLUP_ = 0.26 to r_TY−MGBLUP_ = 0.31 of GBLUP versus MGBLUP, and r_TY−GBLUP_ = 0.31 to r_TY−MGBLUP_ = 0.33 in 2015 (which had a genetic correlation of 0.62 ± 0.19) (Figure 1; Table 2). For MFW we found an increase of PA from r_TY−GBLUP_ = 0.42 to r_TY−MGBLUP_ = 0.55 in the year 2011 in CNJ02 when using GBLUP vs. MGBLUP, which used an additional year of data as an additional response (which had a genetic correlation of 0.93 ± 0.10), and an increase of PA from r_TY−GBLUP_ = 0.28 to r_TY−MGBLUP_ = 0.55 in the year 2012. In the GRYG population in 2014 we found an increase from r_TY−GBLUP_ = 0.36 to r_TY−MGBLUP_ = 0.45 of GBLUP versus MGBLUP, and r_TY−GBLUP_ = 0.34 to r_TY−MGBLUP_ = 0.43 in 2015 (which had shown genetic correlation of 0.93 ± 0.08) (Figure 1; Table 2). In addition, we found no difference in the PA among the univariate models using additive, additive + dominance and/or additive + dominance + epistatic kernels. The epistatic variance component had a trend to be zero across most iterations for both traits, and although the dominance variance component was different than zero, it did not provide an increase in the PA (Figure 1; Table 3).

**Table 3:** By-year comparison of four prediction methods (GBLUP-A, GBLUP-AD, GBLUP-ADE, MGBLUP) based on predictive abilities (and standard deviation) for total yield (TY) and mean fruit weight (MFW) in three biparental populations (CNJ02, *N* = 148; CNJ04, *N* = 67; GRYG, *N* = 351).

**TRAIT**	**Method**	**YEAR**	**POP**	**PAμ**	**PAσ**
TY	A	Y2011	CNJ02	0.124	0.128
TY	AD	Y2011	CNJ02	0.093	0.159
TY	ADE	Y2011	CNJ02	0.092	0.158
TY	MA	Y2011	CNJ02	0.318	0.162
TY	A	Y2012	CNJ02	0.156	0.163
TY	AD	Y2012	CNJ02	0.111	0.198
TY	ADE	Y2012	CNJ02	0.111	0.197
TY	MA	Y2012	CNJ02	0.305	0.203
TY	A	Y2012	CNJ04	0.119	0.232
TY	AD	Y2012	CNJ04	0.045	0.278
TY	ADE	Y2012	CNJ04	0.028	0.281
TY	A	Y2014	GRYG	0.263	0.096
TY	AD	Y2014	GRYG	0.255	0.106
TY	ADE	Y2014	GRYG	0.261	0.095
TY	MA	Y2014	GRYG	0.310	0.096
TY	A	Y2015	GRYG	0.332	0.087
TY	AD	Y2015	GRYG	0.327	0.089
TY	ADE	Y2015	GRYG	0.324	0.090
TY	MA	Y2015	GRYG	0.360	0.093
MFW	A	Y2011	CNJ02	0.420	0.128
MFW	AD	Y2011	CNJ02	0.400	0.141
MFW	ADE	Y2011	CNJ02	0.395	0.135
MFW	MA	Y2011	CNJ02	0.554	0.113
MFW	A	Y2012	CNJ02	0.288	0.136
MFW	AD	Y2012	CNJ02	0.272	0.131
MFW	ADE	Y2012	CNJ02	0.289	0.129
MFW	MA	Y2012	CNJ02	0.517	0.113
MFW	A	Y2012	CNJ04	0.091	0.266
MFW	AD	Y2012	CNJ04	0.026	0.287
MFW	ADE	Y2012	CNJ04	0.001	0.292
MFW	A	Y2014	GRYG	0.361	0.086
MFW	AD	Y2014	GRYG	0.358	0.085
MFW	ADE	Y2014	GRYG	0.358	0.085
MFW	MA	Y2014	GRYG	0.454	0.082
MFW	A	Y2015	GRYG	0.343	0.092
MFW	AD	Y2015	GRYG	0.340	0.092
MFW	ADE	Y2015	GRYG	0.332	0.092
MFW	MA	Y2015	GRYG	0.439	0.083

**Figure 2:**
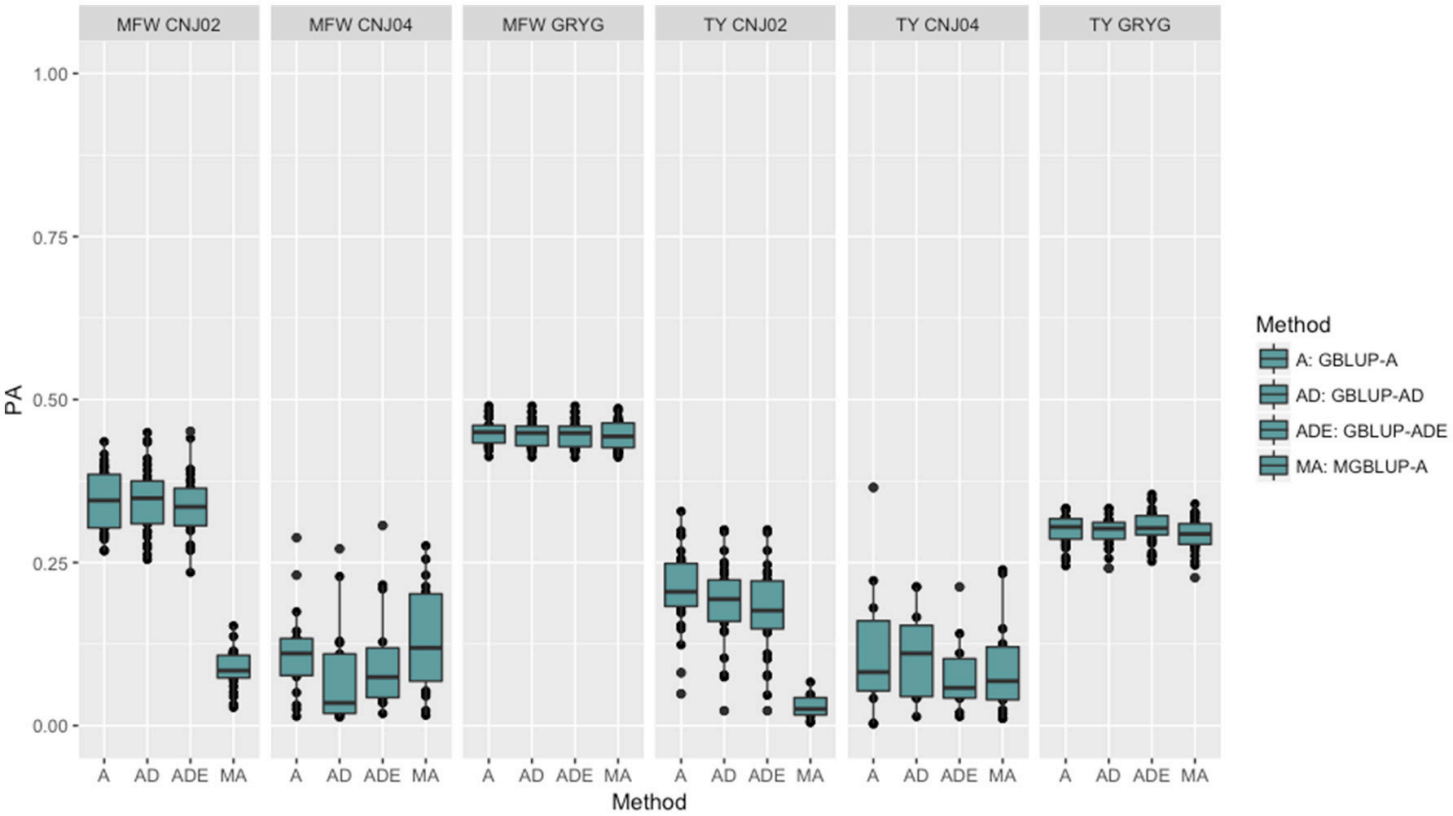
Trait-based comparison between univariate and multivariate genomic best linear unbiased prediction methods (GBLUP and MGBLUP) for mean fruit weight (MFW) and total yield (TY) in three cranberry biparental populations. Methods within boxplots are GBLUP using only additive relationship matrix (GBLUP-A), GBLUP using additive and dominance relationship matrices (GBLUP-AD), GBLUP using additive, dominance and epistatic relationship matrices (GBLUP-ADE), and multivariate GBLUP using only additive relationship matrix (MGBLUP). MGBLUP used both traits to form the multivariate response and the genetic correlation among these responses (low or null genetic correlation scenario in our data).

To look at the effect in PA using multivariate models under a low genetic correlation, we used the across-environment genotype predictions as a response for each trait and population (where MGBLUP uses as multivariate response the predictions for both traits which hold a low genetic correlation in our data). We found no differences between GBLUP-A and MGBLUP across all populations, except for CNJ02 where MGBLUP was notoriously much less accurate to predict the genetic BLUP. For example, in CNJ02 the mean PA for GBLUP was 0.34 whereas for MGBLUP was 0.09 for MFW. For TY we obtained a PA of 0.21 for GBLUP and 0.01 for MGBLUP. In the other populations MGBLUP did as well as GBLUP. For example in GRYG population (biggest population), both GBLUP and MGBLUP had a PA of 0.44 for MFW, and 0.3 for TY (Figure 2).

### Effect of marker density in prediction

**Figure 3:**
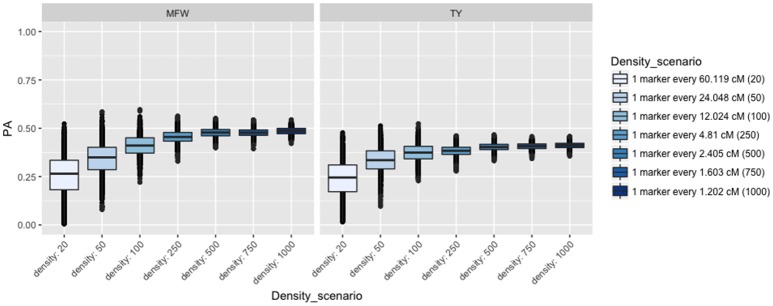
Effect of the marker density on the predictive ability (PA) in GRYG population using across-year estimates adjusted by spatial effects in TY and MFW. One box per trait is displayed (MFW on the left and TY on the right). Within each box a boxplot comparing the different marker densities is shown, from smallest (left) to highest density (right).

To examine the influence of the marker density on the PA, we fitted the univariate GBLUP model by constructing the genomic relationship matrix (**A**_g_) with different number of markers equally spaced and covering the entire genome (Lorenzana and Bernardo, 2009) and used the across-environment genotype predictions as a response. For example, we built the relationship matrix with 20 markers (one marker every 60 cM), 50 (one marker every 24 cM), and so on for 20, 50, 100, 250, 500, 750, 1,000 (covering 1,250 cM). After performing 100 iterations of 5-fold CV, the PA for both traits followed the same linear trend reaching a plateau at about 500 markers (Figure 3). The maximum PA for TY was 0.40 and 0.47 for MFW. We found that addition of markers after 500 markers (i.e., from 750 or 1,000) resulted in only a 0.01 increase of PA in both traits TY and MFW in the three biparental populations. As more markers were used to build the A matrix, the standard error for the PA decreased as well (Figure 3).

### Effect of training population in prediction

**Figure 4:**
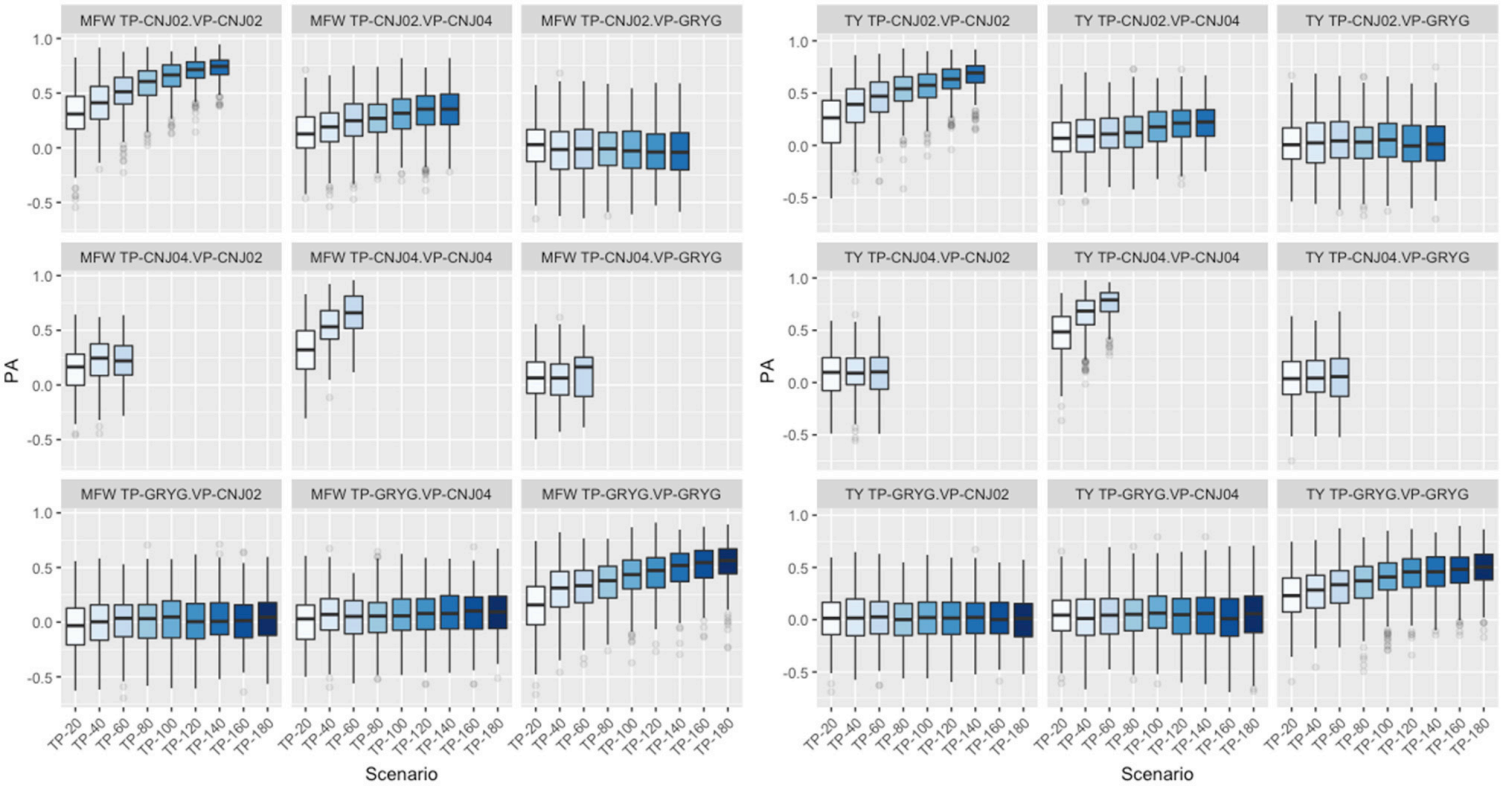
Effect of degree of resemblance on the predictive ability on three biparental populations (CNJ02, CNJ04, and GRYG). The effect on predictive ability (PA) related to the familial relationship between the training population (TP) and validation population (VP) for mean fruit weight (MFW; left box) and total yield (TY; right box).

Following findings by Lorenz and Smith (2015), the effect of resemblance between the TP and VP was examined in the three biparental populations used in the study. We fixed the VP in the GBLUP model and varied the genetic background of the TP. When CNJ02 was fixed as VP and using non-related individuals to CNJ02 (GRYG population) as TP, this yielded the smallest PA. Better PAs were observed when using related half-sib individuals to CNJ02 (CNJ04 population) as the TP. The maximum PA was found when the TP was composed of full-sib individuals from the same VP population CNJ02 as expected. The same tendency in PA was found when CNJ04 and GRYG were fixed as VP, and the best PAs were obtained as the TP were more related to the VP. The increase in TP size was important to increase the PA (Figure 4). This was observed in both traits.

## Discussion

### Genetic correlations

Multivariate BLUP models were originally proposed in animal breeding to model the genetic correlation among traits, longitudinal data and to model genotype by environment interactions in order to exploit the existent correlations in the data (Mrode, 2014; Lee and Van der Werf, 2016). Since the first application of BLUP for multiple trait evaluation by Henderson and Quaas (1976), multiple studies have shown the potential of multivariate mixed models in breeding under classical and genome-assisted approaches (Schaeffer, 1984; Thompson and Meyer, 1986; Burgueño et al., 2012; Jia and Jannink, 2012; Marchal et al., 2016). To test the advantages that multivariate methods could bring to the ongoing GS efforts in American cranberry, we used repeated measures for total yield (TY) and mean fruit weight (MFW) from three biparental populations across different years in the 2011–2015 interval. We presented genetic correlations among years within traits for all biparental populations where each by-environment genotype prediction can be considered as a response, and we found the genetic correlation among years to be high (Table 2). The high genetic correlations between years for MFW (i.e., 0.93) in CNJ02 and GRYG populations were in agreement with breeders observing consistent fruit size along years under commercial production, where uniform management usually results in low genotype by environment (GxE) effects for fruit size (N. Vorsa, personal communication). On the other hand, the genetic correlations between years for CNJ02 and GRYG populations for TY were more variable (i.e., 0.63–0.93), which reflects the a natural phenomenon of quantitative traits such as yield is subject to large genotype by environment (GxE) effects, and in fruit crops a particular physiological phenomenon called “biennial bearing,” which is the incidence of “on” and “off” years of production leads to cyclical yield patterns (Jonkers, 1979; Strik et al., 1991; Curry and Greene, 1993; DeVetter et al., 2013; Schlautman et al., 2015). However, cultural practices and new cultivars have changed or almost removed biennial bearing tendencies in some crops. In cranberry, however, a recently domesticated species, modern cultivars still possess a strong biennial cycle, making genetic evaluation challenging and a long-term process (DeVetter et al., 2013). The fact that the genetic correlation among years (environments) in each biparental population (in both traits) was relatively high, made us compare the univariate and multivariate GS models under the most favorable scenario where an additional response could be used to enhance the PA of a trait displaying a low *h*^2^ for different reasons (i.e., environment, management conditions, etc.).

Additionally, we also calculated the genetic correlation between MFW and TY using across-environment genotype predictions in the three biparental populations (Table 2). The analysis showed that in CNJ02 (*N* = 148) and GRYG (*N* = 351) biparental populations, the genetic correlation between these two traits was equal to zero (considering the standard error), which shows the potential effect in the PA of multivariate GS under a low genetic correlation scenario, where the use of a non-correlated trait does not help and even adds noise to the predictions. For CNJ04 the genetic correlation was different than zero and positive, but with a high SE (Table 2). The fact that the population size of CNJ04 is rather small (*N* = 67) could be an impediment for the correct estimation of the genetic correlation and conclusions based on this small population should be considered carefully.

The fact that the genetic correlation between TY and MFW was practically zero -if we consider the estimate and its standard error- is encouraging for breeding purposes given that this means that selection of large berries does not have a positive or negative effect in the final yield and vice versa. Therefore, our data indicates that high-yielding, large-berry varieties could be successfully developed, which is the case in some modern cranberry cultivars recently developed. Even though, our study does not represent the entire germplasm variability available in breeding programs, our study provides an understanding, based on three-biparental populations, of the genetic correlations among TY and MFW and the genetic correlation among years for such traits in cranberry.

### Model comparison

Genomic selection (GS), first introduced by Meuwissen et al. (2001), has quickly become the preferred MAS method for quantitative traits and is being effectively applied in plant and animal, public or private breeding programs. Perennial fruit crops, on the other hand, have adopted this methodology slower due to particularities of perennial breeding; although these crops could benefit the most given the long development cycle and high-cost associated with fruit crop evaluation (input per genotype evaluated). Moreover, the evaluation of cranberry clones is challenging due to the high-cost involved to evaluating the genotypes under commercial conditions, which require the development of flooding beds (used for water harvesting). Additionally, due to the high cost of developing plantings, cranberry breeding programs rely in low replication designs for the evaluation of a small number of biparental families with a relatively high number of individuals per family (i.e., 300–500). Also, the physical and chemical traits evaluated in cranberry require long evaluation periods (up to 20 years) due to the long time to establish plantings, lengthy juvenility period, and biennial cycling. All the above mentioned challenges make the use of GS techniques very attractive for cranberry breeding to allow the evaluation of a larger number of families by reducing the number of individuals per family and shortening the selection cycles. Thus, we not only evaluated the efficiency GS in cranberry, but also compared univariate and multivariate GBLUP methods to improve breeding efforts.

GBLUP has been already shown to be superior to classical MAS, which uses only the most significant markers from traditional QTL studies (Lorenzana and Bernardo, 2009; Heffner et al., 2011). Since the initial proposal of using all the markers through the computation of the genomic relationship matrix, the development and inclusion of dominance and epistastic kernels in the univariate models and multivariate models has been tested showing limited advantage of such additional kernels. In this study, we found that the inclusion of dominance and epistatic kernels did not yield higher accuracies than the regular GBLUP model that incorporates only the additive kernel, which is consistent with results from other research groups (Su et al., 2012; Muñoz et al., 2014). From our perspective, this phenomenon follows the laws of parsimony, where models that better explain the data are not necessarily the best prediction machines (Hastie et al., 2009). In addition, the fact that a rather small number of populations is presented in this research makes the resolution to estimate and exploit non-additive effects very low. Also, most popular methods to calculate the dominance and epistatic kernels yield relationship matrices are not completely orthogonal to the additive relationship matrix, making their effectiveness for prediction questionable, which has led different research groups to investigate the topic and propose other orthogonal methods (Xiang et al., 2018). The purpose of comparing the regular GBLUP against GBLUP-AD in our study was driven by the fact that half- and full-sib populations share $\frac{1}{4}$ and $\frac{1}{2}$ of the dominance variation ($\sigma_{\text{D}}^{2}$). In our hypothesis, we expected these terms to be different than zero and maybe contribute to an increase in PA proportional to the explained variation. The inclusion of dominance kernels indeed yielded variance component estimators different than zero, which was according to our expectations, but this did not increase the PA compared to the only-additive model. We found the maximum PA for both traits to be nearly the square root of the h^2^ as expected given that the PA and the h^2^ are intrinsically connected. We found the same relationship in the PA for TY and MFW (Tables 1, 3).

Selection of elite genotypes is commonly based on a combination of several traits of economic importance, which might be genetically correlated. A multiple trait evaluation is a popular methodology to evaluate individuals accounting for relationships among traits (Mrode, 2014). Extensive animal breeding literature using multi-trait models has been generated using multi-trait pedigree-based BLUP (MPBLUP) (Schaeffer, 1984; Thompson and Meyer, 1986; Mrode, 2014). With the massive availability of markers, multi-trait models using genomic information (MGBLUP) are now feasible to model and non-model organisms where pedigree information is not robust. In order to test the advantages of multivariate mixed models for GS in current cranberry breeding efforts, we compared the univariate and multivariate versions of the GBLUP model.

Simulation studies have shown that an increase in PA from 3 to 14% can be achieved when genetic correlations among responses range from 0.25 to 0.75 (Calus and Veerkamp, 2011). In addition, Jia and Jannink (2012) showed that multivariate GS could increased the PA for a low-heritability trait when a high-heritability and correlated trait is available (Jia and Jannink, 2012; Mrode, 2014). The research previously cited has found higher PA for the multi-trait approach than single-trait GS when phenotypes are not available on all individuals and traits. In other crops such as oil palm, multivariate genomic models have increased the accuracy of progeny tests (Marchal et al., 2016). When comparing the MGBLUP against the univariate GBLUP using additional years of data as additional responses (by-environment genotype predictions), we found a clear increase in the PA because of the high genetic correlation of the genotypes with themselves in additional environments (i.e., 0.93 in TY and MFW for CNJ02 and GRYG). We found instances where an increase of 0.03–0.06 units (8–17% increase) in PA was observed when the responses had a genetic correlation of 0.63 (Figure 1; Tables 2, 3). When the genetic correlation of the responses was higher (i.e., 0.93) we found increases of PA of 0.15–0.19 (25–156% increases depending on the trait) (Tables 2, 3; Figure 1). On the other hand, when using the across-environment genotype predictions from TY and MFW as multivariate response, we found a genetic correlation of zero for CNJ02 and GRYG populations (−0.01 ± 0.20 and −0.29 ± 0.38 respectively), which allowed us to observe the effect in PA when the responses have a null genetic correlation. We observed that MGBLUP did not increase the PA with respect to univariate GBLUP in all populations.

In general, the PAs and h^2^'s for both traits in the analyses using by-environment genotype predictions were low to intermediate due to the low replication within environments commonly used in cranberry breeding (Technow et al., 2014; Zhao et al., 2015). When using across-environment genotype predictions as input for GS models, we found higher PA's as expected when the level of replication increases (Figure 3). For example, Technow et al. (2014) presented PAs of up to 0.9 for TY in maize when using across-environment genetic estimates (from 20 locations during 14 years 131 environments), which allows for an accurate estimation of the general BVs for genotypes. Zhao et al. (2015) found a PA of 0.89 for TY in wheat commercial and breeding lines evaluated in 11 environments in a p-rep design. The greater the number of environments used (replication), the greater the across-environment *h*^2^ is and as consequence the PAs when using such across the environment estimates (Zhao et al., 2015). In this study we found an important increase in the PA when performing GS using estimates across environments in both populations with more than 1 year of data (CNJ02 and GRYG) for both traits TY and MFW.

### Effect of marker density in prediction

Various factors including the resemblance between TP and VP, TP size, marker density, heritability, magnitude of LD, trait architecture, and the interaction among all of them appear to be the principal forces driving PA. Marker density is by far one of the most studied factors determining PA in GS experiments. The consensus is that a higher number of markers usually yield higher PA reaching a plateau depending on the architecture of the trait, the number of individuals in the TP, the size of the genome and linkage disequilibrium. Lorenzana and Bernardo (2009) showed such relationship in maize, barley, and *Arabidopsis* and concluded that predictions became more accurate as the number of individuals and number of markers increased. Such tendency holds given that the more markers covering the genome of the population, the higher the probability of having a marker in LD with the causal variant (Habier et al., 2013). Therefore, the number of markers needed is proportional to the diversity present in the VP and TP (LD). In the present study, we found that PA increased as the number of markers increased, but only 500–750 markers were required to reach the maximum PA within biparental populations. The low number of markers required to reach a maximum PA reflect the high degree of genetic structure present in biparental populations which are in full linkage disequilibrium (Figure 2). This observation is typical in biparental populations, but not in panels of diversity where LD can break at very short genetic and physical distances. The low number of markers required to reach the maximum PA agrees with the LD decay estimated in this study of ~18 cM (at *r*^2^ = 0.2) on average among the three biparental populations, which confirming that few markers are required to have enough LD with the causal variant to capture marker effects in the GS model in biparental populations (Lorenzana and Bernardo, 2009).

### Effect of TP-VP resemblance in prediction

The effect of resemblance between TPs and VPs on the PA has been described by several research groups. For example, Riedelsheimer et al. (2013), highlighted an important feature of GS, which implies that a higher genetic resemblance often result in greater accuracies. A similar phenomenon was found by Lorenz and Smith (2015) who observed that adding genetically distant individuals to the TPs resulted in a reduction in the PA in barley populations. However, Lorenz and Smith (2015) suggested that their results could be conditional on the low marker density used (342 SNPs). Still, their findings suggest that plant breeding programs could benefit from focusing on good phenotyping of smaller TPs closely related to the selection candidates rather than large and diverse TPs. This is particularly important in cranberry breeding, which relies on large-sized biparental populations that are often closely related to each other given the low number of elite parents currently used.

In this study, the three full-sib populations used had different degrees of relationship; GRYG had a distant relationship with CNJ02 and CNJ04, whereas CNJ02 and CNJ04 were half-sibs. When the GBLUP model was used for TY and we fixed CNJ02 as the VP and varied the genetic background of the TP using non-related individual from GRYG (at different TP sizes), half-sib individuals from CNJ04, and full-sib individuals from the CNJ02, we found greater PAs as the TP was more related to the VP (Bassi et al., 2016). The same PA tendency was found when CNJ04 and GRYG were fixed as VP (Figure 4). These results were similar to those found by Lorenz and Smith (2015) where higher resemblances resulted in greater accuracies when predicting an individual in the following order: full-sibs, half-sibs, and no relationship. Increasing the TP size also increased the PA in all scenarios without reaching a plateau with the available population sizes. Plant breeding programs take advantage of these relationships by carefully planning the new-generation crosses to reuse TPs from previous generations as long as the TP and VP share some relationship and new individuals are added to the TP to retrain the models. This strategy can be easily implemented in recurrent selection schemes, which are the base of most breeding programs.

## Conclusions

GS has gained popularity in plant and animal breeding due to its straightforward use within ongoing breeding programs. Unfortunately, minor fruit crops have hardly explored the potential of GS. We found GS to be effective in cranberry biparental populations using the GBLUP approach and reaching its maximum PA with relatively few markers (~500–750) due to the full LD typically present in biparental populations. This implies that in structured populations (i.e., biparental), such as those used in the cranberry breeding programs, a medium marker density is enough to reach maximum PA. The conformation of the TP and its resemblance with the VP were shown to be decisive factors in achieving maximum PA. In addition, we were particularly interested in testing the advantages of using multivariate compared to univariate GBLUP, and the former was shown to provide a positive impact in the PA when the genetic correlation among the responses was high (i.e., 0.6), and to have a negative effect in the PA when the correlation was close to zero. We conclude that the use of multivariate methods to select plants simultaneously for different traits and to predict traits of low heritability should be considered in cranberry breeding, as well as in other fruit crops and understudied species.

## Author contributions

GC-P, BS, and LD-G performed the analysis and write the manuscript. EG, JP, and JJ-C grew the experimental units and maintained the populations and review the manuscript. NV, MI, JZ, and GC-P planned the research and write the manuscript. All coauthors reviewed and approved the final version of this manuscript.

### Conflict of interest statement

The authors declare that the research was conducted in the absence of any commercial or financial relationships that could be construed as a potential conflict of interest. The reviewer PRM and handling editor declared their shared affiliation at the time of the review.

## Supplementary material

Supplementary File 1 Phenotypic and genotypic data.

Supplementary File 2 Spatial modeling and heritabilities.

Supplementary File 3 Cross-validations with by-year BLUPs.

Supplementary File 4 Effect of marker density in PA.

Supplementary File 5 Cross-validation with across years BLUPs.

## Abbreviations

CV: cross-validation
GBLUP: genomic best linear unbiased predictor
GBS: genotyping-by-sequencing
LD: linkage disequilibrium
LG: linkage group
MGBLUP: multivariate genomic BLUP
MFW: mean fruit weight
PA: predictive ability
QTL: quantitative trait loci
SNP: single nucleotide polymorphism
SSR: simple sequence repeat
TP: training population
TY: total yield
VP: validation population.
