# Supplementary material for: Multivariate GBLUP Improves Accuracy of Genomic Selection for Yield and Fruit Weight in Biparental Populations of Vaccinium macrocarpon Ait
Source: Front Plant Sci. 2018 Sep 12;9:1310. doi: 10.3389/fpls.2018.01310 (PMC6144488; doi:10.3389/fpls.2018.01310)
Supplement: Supplementary File 2 — Spatial modeling and heritabilities. [file Table_2.docx]

**Supplementary File 2**

library(sommer)

#library(lattice)

library(agridat)

setwd("~/Desktop/VMGSIC/new version 2018/pheno and geno")

load(file="GSpaper.RData")

head(grig.pheno)

head(cnj02.pheno)

head(grig.pheno)

boxplot(grig.pheno$yield)

library(agridat)

desplot(yield~Row*Col|year, data=grig.pheno)

desplot(yield~Row*Col|year, data=cnj02.pheno)

desplot(yield~Row*Col|year, data=cnj04.pheno)

traits <- c("yield","wpfruit")

############################################################

## blups for GS by year and genetic correlations among years

############################################################

grig.pheno$yield[which(grig.pheno$yield < 5)] <- NA

cnj02.pheno$yield[which(cnj02.pheno$yield < 5)] <- NA

cnj04.pheno$yield[which(cnj04.pheno$yield < 5)] <- NA

grig.pheno$wpfruit[which(grig.pheno$wpfruit < .1)] <- NA

cnj02.pheno$wpfruit[which(cnj02.pheno$wpfruit < .1)] <- NA

cnj04.pheno$wpfruit[which(cnj04.pheno$wpfruit < .1)] <- NA

for(o in 1:2){

yearblups <- list()

for(itrait in traits){ # itrait <- traits[1]

print(itrait)

# ss <- spl2D(grig.pheno, x.coord = "Row", y.coord = "Col", by="yearf")

ranran <- "~at(yearf):id + at(yearf):Rowf + at(yearf):Colf + spl2D(Row,Col, at=yearf) "

# ranran <- paste(c(ranran,ss$funny), collapse = " + ")

fixedf <- as.formula(paste(itrait,"~yearf"))

mix.grig <- mmer2(fixedf,

random = as.formula(ranran),

rcov=~at(yearf):units, #draw = TRUE, iters = 40,

iters = 40,

data=grig.pheno)

print(paste("grig model converged:", mix.grig$convergence))

# summary(mix.grig)

# remove model-based outliers

grig.pheno[which(!is.na(grig.pheno[,itrait])), "res"] <- scale(residuals(mix.grig))

grig.pheno[which(grig.pheno[,"res"] > 3.5 | grig.pheno[,"res"] < -3.5),itrait] <- NA

res <- names(mix.grig$u.hat)[grep("id",names(mix.grig$u.hat))]

blup1 <- do.call(cbind,mix.grig$u.hat[res])

colnames(blup1) <- gsub(":.*","",res); blup1 <- as.data.frame(blup1)

blup1$id <- gsub(".*:id","",rownames(blup1))

blup1[which(blup1==0, arr.ind = TRUE)] <- NA

## quality check

# names(ss$newdat)

# names(mix.grig$Zus)

vm0 <- variogram(mix.grig, xcoor = "Row", ycoor = "Col", by="yearf")

lapply(vm0, plot)

####

####

#### CNJ02

####

####

#ss <- spl2D(cnj02.pheno, x.coord = "Row", y.coord = "Col", by="yearf")

ranran <- "~at(yearf):id + at(yearf):Rowf + at(yearf):Colf + spl2D(Row,Col, at=yearf)"

#ranran <- paste(c(ranran,ss$funny), collapse = " + ")

fixedf <- as.formula(paste(itrait,"~yearf"))

mix.cnj02 <- mmer2(fixedf,

random = as.formula(ranran),

rcov=~at(yearf):units,

iters = 40,

data=cnj02.pheno)

print(paste("cnj02 model converged:", mix.cnj02$convergence))

# remove model-based outliers

cnj02.pheno[which(!is.na(cnj02.pheno[,itrait])), "res"] <- scale(residuals(mix.cnj02))

cnj02.pheno[which(cnj02.pheno[,"res"] > 3.5 | cnj02.pheno[,"res"] < -3.5),itrait] <- NA

res <- names(mix.cnj02$u.hat)[grep("id",names(mix.cnj02$u.hat))]

blup2 <- do.call(cbind,mix.cnj02$u.hat[res])

colnames(blup2) <- gsub(":.*","",res); blup2 <- as.data.frame(blup2)

blup2$id <- gsub(".*:id","",rownames(blup2))

blup2[which(blup2==0, arr.ind = TRUE)] <- NA

## quality check

vm0 <- variogram(mix.cnj02, xcoor = "Row", ycoor = "Col", by="yearf")

lapply(vm0, plot)

# cnj02.pheno$res <- NA

# cnj02.pheno$res[which(!is.na(cnj02.pheno[,itrait]))] <- residuals(mix.cnj02)

# print(desplot(res~Row*Col|year, data=cnj02.pheno))

# print(xyplot(res~Row|yearf, data=cnj02.pheno))

# print(xyplot(res~Col|yearf, data=cnj02.pheno))

####

####

#### CNJ04

####

####

#ss <- spl2D(cnj04.pheno, x.coord = "Row", y.coord = "Col", by="yearf")

ranran <- "~at(yearf):id + at(yearf):Rowf + at(yearf):Colf + spl2D(Row,Col, at=yearf) "

#ranran <- paste(c(ranran,ss$funny), collapse = " + ")

fixedf <- as.formula(paste(itrait,"~yearf"))

mix.cnj04 <- mmer2(yield~yearf,

random = as.formula(ranran),

rcov=~at(yearf):units,

grouping = ss$newdat,iters = 40,

data=cnj04.pheno)

print(paste("cnj04 model converged:", mix.cnj04$convergence))

# remove model-based outliers

cnj04.pheno[which(!is.na(cnj04.pheno[,itrait])), "res"] <- scale(residuals(mix.cnj04))

cnj04.pheno[which(cnj04.pheno[,"res"] > 3.5 | cnj04.pheno[,"res"] < -3.5),itrait] <- NA

res <- names(mix.cnj04$u.hat)[grep("id",names(mix.cnj04$u.hat))]

blup3 <- do.call(cbind,mix.cnj04$u.hat[res])

colnames(blup3) <- gsub(":.*","",res); blup3 <- as.data.frame(blup3)

blup3$id <- gsub(".*:id","",rownames(blup3))

## quality check

vm0 <- variogram(mix.cnj04, xcoor = "Row", ycoor = "Col", by="yearf")

lapply(vm0, plot)

blup3[which(blup3==0, arr.ind = TRUE)] <- NA

# cnj04.pheno$res <- NA

# cnj04.pheno$res[which(!is.na(cnj04.pheno[,itrait]))] <- residuals(mix.cnj04)

# print(desplot(res~Row*Col|year, data=cnj04.pheno))

# print(xyplot(res~Row|yearf, data=cnj04.pheno))

# print(xyplot(res~Col|yearf, data=cnj04.pheno))

yearblups[[itrait]] <- list(grig=blup1, cnj02=blup2, cnj04=blup3)

}

}

for(u in 1:3){

lapply(yearblups$yield, function(x){boxplot(x[[u]],main=paste("pop",u,"yield"))})

lapply(yearblups$wpfruit, function(x){boxplot(x[[u]],main=paste("pop",u,"wpfruit"))})

}

head(yearblups$yield$grig)

plot(yearblups$yield$grig[,-3])

############################################################

## Numeric marker matrices and A.mats

############################################################

rownames(grig.geno) <- grig.geno$Locus

grig.geno[which(grig.geno == "--", arr.ind = TRUE)] <- NA

M.grig <- atcg1234(t(grig.geno[,-c(1:5)]))

M.grig[1:4,1:4]

A.grig <- A.mat(M.grig)

D.grig <- D.mat(M.grig)

E.grig <- E.mat(M.grig)

image(A.grig)

rownames(cnj02.geno) <- cnj02.geno$Locus

cnj02.geno[which(cnj02.geno == "--", arr.ind = TRUE)] <- NA

M.cnj02 <- atcg1234(t(cnj02.geno[,-c(1:5)]))

M.cnj02[1:4,1:4]

A.cnj02 <- A.mat(M.cnj02)

D.cnj02 <- D.mat(M.cnj02)

E.cnj02 <- E.mat(M.cnj02)

image(A.cnj02)

rownames(cnj04.geno) <- cnj04.geno$Locus

cnj04.geno[which(cnj04.geno == "--", arr.ind = TRUE)] <- NA

M.cnj04 <- atcg1234(t(cnj04.geno[,-c(1:5)]))

M.cnj04[1:4,1:4]

A.cnj04 <- A.mat(M.cnj04)

D.cnj04 <- D.mat(M.cnj04)

E.cnj04 <- E.mat(M.cnj04)

image(A.cnj04)

A <- list(grig=A.grig, cnj02=A.cnj02, cnj04=A.cnj04)

D <- list(grig=D.grig, cnj02=D.cnj02, cnj04=D.cnj04)

E <- list(grig=E.grig, cnj02=E.cnj02, cnj04=E.cnj04)

############################################################

## Inspect univariate models, year-trait h2's

############################################################

traitLIST <- list()

for(itrait in traits){ # itrait <- traits[2]

data0 <- yearblups[[itrait]]

pops <- c("grig","cnj02","cnj04")

# pop

popLIST <- list()

for(u in pops){ # u <- pops[1]

prov.pop <- data0[[u]]

colnames(prov.pop)[which(colnames(prov.pop) != "id")] <- paste("Y",colnames(prov.pop)[which(colnames(prov.pop) != "id")],sep="")

prov.pop[which(prov.pop == 0, arr.ind = TRUE)] <- NA

A.pop <- A[[u]]

common.pop <- intersect(rownames(A.pop), prov.pop$id)

prov.pop <- prov.pop[which(prov.pop$id %in% common.pop),]

# year

ys <- setdiff(colnames(prov.pop), "id")

yslist <- list()

for(w in ys){ # w <- ys[2]

fixf <- as.formula(paste(w,"~1"))

mix.pop <- mmer2(fixf,

random = ~ g(id),

rcov=~ units,iters = 40,

G=list(id=A.pop),

data=prov.pop)

summary(mix.pop)

yslist[[w]] <- pin(mix.pop, h2~V1/(V1+V2)) # year1 heritabilities

}

ysdata <- data.frame(do.call(rbind,yslist))

ysdata$year <- names(yslist)

ysdata$pop <- u

ysdata$trait <- itrait

popLIST[[u]] <- ysdata

}

popdata <- as.data.frame(do.call(rbind, popLIST))

traitLIST[[itrait]] <- popdata

}

traitdata <- as.data.frame(do.call(rbind, traitLIST))

traitdata

write.csv(traitdata,file=file.path(outdir,"h2s.csv"))

traitdata[which(traitdata$Estimate > 0.1),]

# once bad years are identified let's delete them so they

# are not used in posterior calculations (multivariate models)

cnj02.pheno[which(cnj02.pheno$yearf == "Y2013"),c("wpfruit")] <- NA

cnj02.pheno[which(cnj02.pheno$yearf == "Y2013"),c("yield")] <- NA

cnj04.pheno[which(cnj04.pheno$yearf == "Y2011"),c("wpfruit")] <- NA

cnj04.pheno[which(cnj04.pheno$yearf == "Y2014"),c("wpfruit")] <- NA

cnj04.pheno[which(cnj04.pheno$yearf == "Y2011"),c("yield")] <- NA

cnj04.pheno[which(cnj04.pheno$yearf == "Y2014"),c("yield")] <- NA

############################################################

## blups for genetic correlations among traits

############################################################

acrossblups <- list()

for(itrait in traits){ # itrait <- traits[1]

print(itrait)

poplist <- list()

fixedf <- as.formula(paste(itrait,"~1"))

subdata <- grig.pheno

subdata <- droplevels(subdata[which(!is.na(subdata[,itrait])),])

#ss <- spl2D(subdata, x.coord = "Row", y.coord = "Col", by="yearf")

ranran <- "~id + yearf + at(yearf):Rowf + at(yearf):Colf + spl2D(Row,Col, at=yearf)"

#ranran <- paste(c(ranran,ss$funny), collapse = " + ")

fixedf <- as.formula(paste(itrait,"~yearf"))

print("grig")

mix.grig <- mmer2(fixedf,

random = as.formula(ranran),

rcov=~at(yearf):units,

grouping = ss$newdat, iters = 40,

data=subdata)

print(paste("grig model converged:", mix.grig$convergence))

poplist[["grig"]] <- mix.grig$u.hat$id

subdata <- cnj02.pheno

subdata <- droplevels(subdata[which(!is.na(subdata[,itrait])),])

#ss <- spl2D(subdata, x.coord = "Row", y.coord = "Col", by="yearf")

ranran <- "~id + yearf+ at(yearf):Rowf + at(yearf):Colf + spl2D(Row,Col, at=yearf)"

#ranran <- paste(c(ranran,ss$funny), collapse = " + ")

fixedf <- as.formula(paste(itrait,"~yearf"))

print("cnj02")

mix.cnj02 <- mmer2(fixedf,

random = as.formula(ranran),

rcov=~at(yearf):units,iters = 40,

grouping = ss$newdat,

data=subdata)

print(paste("cnj02 model converged:", mix.cnj02$convergence))

poplist[["cnj02"]] <- mix.cnj02$u.hat$id

subdata <- cnj04.pheno

subdata <- droplevels(subdata[which(!is.na(subdata[,itrait])),])

#ss <- spl2D(subdata, x.coord = "Row", y.coord = "Col", by="yearf")

ranran <- "~id + yearf + at(yearf):Rowf + at(yearf):Colf + spl2D(Row,Col, at=yearf) "

#ranran <- paste(c(ranran,ss$funny), collapse = " + ")

fixedf <- as.formula(paste(itrait,"~yearf"))

print("cnj04")

mix.cnj04 <- mmer2(fixedf,

random = as.formula(ranran),

rcov=~at(yearf):units,iters = 40,

grouping = ss$newdat,

data=subdata)

print(paste("cnj04 model converged:", mix.cnj04$convergence))

poplist[["cnj04"]] <- mix.cnj04$u.hat$id

acrossblups[[itrait]] <- poplist

}

acrossblups0<- list()

pops <- c("grig","cnj02","cnj04")

for(i in pops){ # i <- pops[1]

common <- Reduce(intersect, lapply(acrossblups,function(x){rownames(x[[i]])}))

prov <- as.data.frame(do.call(cbind,lapply(acrossblups, function(x){x[[i]][common,]})))

prov$id <- gsub(".*id","",rownames(prov))

acrossblups0[[i]] <- prov

}

############################################################

## calculate genetic correlations among traits

############################################################

# griglesky

common.grig <- intersect(rownames(A.grig), acrossblups0$grig$id)

prov.grig <- acrossblups0$grig

prov.grig <- prov.grig[which(prov.grig$id %in% common.grig),]

prov.grig[which(prov.grig==0, arr.ind = TRUE)] <- NA

mixm.grig <- mmer2(cbind(yield,wpfruit)~1,

random = ~ us(trait):g(id),

rcov=~us(trait):units,

G=list(id=A.grig), draw = TRUE, init.equal = F,

na.method.Y = "exclude",

data=prov.grig)

summary(mixm.grig)

cov2cor(mixm.grig$var.comp$`g(id)`)

pin(mixm.grig, h2~V1/(V1+V4))

pin(mixm.grig, h2~V3/(V3+V6))

pin(mixm.grig, gen.cor ~ V2 / sqrt(V1*V3))

plot(prov.grig[,1:2])

# cnj02

common.cnj02 <- intersect(rownames(A.cnj02), acrossblups0$cnj02$id)

prov.cnj02 <- acrossblups0$cnj02

prov.cnj02 <- prov.cnj02[which(prov.cnj02$id %in% common.cnj02),]

prov.cnj02[which(prov.cnj02==0, arr.ind = TRUE)] <- NA

mixm.cnj02 <- mmer2(cbind(yield,wpfruit)~1,

random = ~ us(trait):g(id),

rcov=~us(trait):units,

G=list(id=A.cnj02),

na.method.Y = "exclude",

data=prov.cnj02)

summary(mixm.cnj02)

cov2cor(mixm.cnj02$var.comp$`g(id)`)

pin(mixm.cnj02, h2~V1/(V1+V4))

pin(mixm.cnj02, h2~V3/(V3+V6))

pin(mixm.cnj02, gen.cor ~ V2 / sqrt(V1*V3))

plot(prov.cnj02[,1:2])

# cnj04 (based in a single year)

common.cnj04 <- intersect(rownames(A.cnj04), acrossblups0$cnj04$id)

prov.cnj04 <- acrossblups0$cnj04

prov.cnj04 <- prov.cnj04[which(prov.cnj04$id %in% common.cnj04),]

prov.cnj04[which(prov.cnj04==0, arr.ind = TRUE)] <- NA

mixm.cnj04 <- mmer2(cbind(yield,wpfruit)~1,

random = ~ us(trait):g(id),

rcov=~us(trait):units,

na.method.Y = "exclude",

G=list(id=A.cnj04),

data=prov.cnj04)

summary(mixm.cnj04)

cov2cor(mixm.cnj04$var.comp$`g(id)`)

pin(mixm.cnj04, h2~V1/(V1+V4))

pin(mixm.cnj04, h2~V3/(V3+V6))

pin(mixm.cnj04, gen.cor ~ V2 / sqrt(V1*V3))

plot(prov.cnj04[,1:2])

boxplot(prov.cnj04[,1:2])

traitcors <- data.frame(Population=c("GRIG","CNJ02","CNJ04"),

rbind(

pin(mixm.grig, gen.cor ~ V2 / sqrt(V1*V3)),

pin(mixm.cnj02, gen.cor ~ V2 / sqrt(V1*V3)),

pin(mixm.cnj04, gen.cor ~ V2 / sqrt(V1*V3))

)

)

#write.csv(traitcors,file=file.path(outdir,"trait_cors.csv"))

############################################################

## calculate genetic correlations among years for each trait

############################################################

traitdata

tres <-list()

for(itrait in traits){ # itrait <- traits[1]

print(itrait)

data0 <- yearblups[[itrait]]

# griglesky

prov.grig <- data0$grig

colnames(prov.grig)[which(colnames(prov.grig) != "id")] <- paste("Y",colnames(prov.grig)[which(colnames(prov.grig) != "id")],sep="")

prov.grig[which(prov.grig == 0, arr.ind = TRUE)] <- NA

common.grig <- intersect(rownames(A.grig), prov.grig$id)

prov.grig <- prov.grig[which(prov.grig$id %in% common.grig),]

ys <- setdiff(colnames(prov.grig), "id")

fixf <- as.formula(paste("cbind(",paste(ys, collapse = ","),")~1"))

mixy.grig <- mmer2(fixf,

random = ~ us(trait):g(id),

rcov=~us(trait):units,

G=list(id=A.grig),

data=prov.grig,

na.method.Y = "exclude")

summary(mixy.grig)

cov2cor(mixy.grig$var.comp$`g(id)`)

pp1 <- as.data.frame(rbind(

pin(mixy.grig, h2~V1/(V1+V4)), # year1 heritabilities

pin(mixy.grig, h2~V3/(V3+V6)), # year2 heritabilites

pin(mixy.grig, r~V2/sqrt(V1*V3)) # year2 heritabilites

))

pp1$param <- c("h2.2014","h2.2015","r")

pp1$trait <- itrait

pp1$pop <- "grig"

#plot(prov.grig[,-3])

# cnj02

prov.cnj02 <- data0$cnj02#[,-1]

plot(prov.cnj02[,-4])

colnames(prov.cnj02)[which(colnames(prov.cnj02) != "id")] <- paste("Y",colnames(prov.cnj02)[which(colnames(prov.cnj02) != "id")],sep="")

prov.cnj02[which(prov.cnj02 == 0, arr.ind = TRUE)] <- NA

common.cnj02 <- intersect(rownames(A.cnj02), prov.cnj02$id)

prov.cnj02 <- prov.cnj02[which(prov.cnj02$id %in% common.cnj02),]

ys <- setdiff(colnames(prov.cnj02), c("id","Y2013")) #

fixf <- as.formula(paste("cbind(",paste(ys, collapse = ","),")~1"))

mixy.cnj02 <- mmer2(fixf,

random = ~ us(trait):g(id),

rcov=~us(trait):units,

G=list(id=A.cnj02),

data=prov.cnj02, iters = 100, #draw = TRUE,

init.equal = F,tolpar = 1e-10,

na.method.Y = "exclude")

summary(mixy.cnj02)

cov2cor(mixy.cnj02$var.comp$`g(id)`)

# pin(mixy.cnj02, h2~V1/(V1+V7)) # year1 heritabilities

# pin(mixy.cnj02, h2~V4/(V4+V10)) # year2 heritabilites

# pin(mixy.cnj02, h2~V6/(V6+V12)) # year2 heritabilites

pp2 <- as.data.frame(rbind(

pin(mixy.cnj02, h2~V1/(V1+V4)), # year1 heritabilities

pin(mixy.cnj02, h2~V3/(V3+V6)) ,# year2 heritabilites

pin(mixy.cnj02, r~V2/sqrt(V1*V3)) # year2 heritabilites

))

pp2$param <- c("h2.2014","h2.2015","r")

pp2$trait <- itrait

pp2$pop <- "cnj02"

#plot(prov.cnj02[,-4])

### cnj04 (only had one year with real h2)

# prov.cnj04 <- data0$cnj04#[,-3]

# colnames(prov.cnj04)[which(colnames(prov.cnj04) != "id")] <- paste("Y",colnames(prov.cnj04)[which(colnames(prov.cnj04) != "id")],sep="")

# prov.cnj04[which(prov.cnj04 == 0, arr.ind = TRUE)] <- NA

# common.cnj04 <- intersect(rownames(A.cnj04), prov.cnj04$id)

# prov.cnj04 <- prov.cnj04[which(prov.cnj04$id %in% common.cnj04),]

# ys <- setdiff(colnames(prov.cnj04), "id")

# fixf <- as.formula(paste("cbind(",paste(ys, collapse = ","),")~1"))

#

# mixy.cnj04 <- mmer2(fixf,

# random = ~ us(trait):g(id),

# rcov=~diag(trait):units,

# G=list(id=A.cnj04),

# data=prov.cnj04, iters = 10,

# na.method.Y = "exclude")

#

# summary(mixy.cnj04)

# cov2cor(mixy.cnj04$var.comp$`g(id)`)

#

# pin(mixy.cnj04, h2~V1/(V1+V7)) # year1 heritabilities

# pin(mixy.cnj04, h2~V4/(V4+V10)) # year2 heritabilites

# pin(mixy.cnj04, h2~V6/(V6+V12)) # year2 heritabilites

#

# pin(mixy.cnj04, h2~V1/(V1+V4)) # year1 heritabilities

# pin(mixy.cnj04, h2~V3/(V3+V6)) # year2 heritabilites

# plot(prov.cnj04[,-3])

tres[[itrait]] <- as.data.frame(rbind(pp1,pp2))

}

Ycors <- as.data.frame(do.call(rbind,tres))

Ycors

Ycors2 <- Ycors[which(Ycors$param == "r"),]

write.csv(Ycors2,file=file.path(outdir,"year_cors.csv"))

outdir <- "~/Desktop/VMGSIC/new version 2018/pheno and geno"

save.image(file.path(outdir,"GSpaper_01_results.RData"))

#load(file.path(outdir,"GSpaper_01_results.RData"))
