# Supplementary material for: Multivariate GBLUP Improves Accuracy of Genomic Selection for Yield and Fruit Weight in Biparental Populations of Vaccinium macrocarpon Ait
Source: Front Plant Sci. 2018 Sep 12;9:1310. doi: 10.3389/fpls.2018.01310 (PMC6144488; doi:10.3389/fpls.2018.01310)
Supplement: Supplementary File 3 — Cross-validations with by-year BLUPs. [file Table_3.docx]

**Supplementary File 3**

###########################################

## cross validation for GS by year

## 5 fold

## MGBLUP uses data from the additional year

###########################################

rm(list=ls())

# "~/Desktop/VMGSIC/new version 2018/pheno and geno"

load("~/Desktop/VMGSIC/new version 2018/pheno and geno/GSpaper_02_results.RData")

library(sommer)

traitLIST <- list()

for(itrait in traits){ # itrait <- traits[2]

print(itrait)

data0 <- yearblups[[itrait]]

pops <- c("grig","cnj02","cnj04")

# pop

popLIST <- list()

for(u in pops){ # u <- pops[3]

print(u)

prov.pop <- data0[[u]]

colnames(prov.pop)[which(colnames(prov.pop) != "id")] <- paste("Y",colnames(prov.pop)[which(colnames(prov.pop) != "id")],sep="")

if(u=="cnj02"){prov.pop <- prov.pop[,setdiff(colnames(prov.pop),"Y2013")]}

if(u=="cnj04"){prov.pop <- prov.pop[,setdiff(colnames(prov.pop),c("Y2011","Y2014"))]}

prov.pop[which(prov.pop == 0, arr.ind = TRUE)] <- NA

A.pop <- A[[u]]

D.pop <- D[[u]]

E.pop <- E[[u]]

common.pop <- intersect(rownames(A.pop), prov.pop$id)

prov.pop <- prov.pop[which(prov.pop$id %in% common.pop),]

prov.pop$idd <- prov.pop$id

prov.pop$ide <- prov.pop$id

# year

ys <- setdiff(colnames(prov.pop), c("id","idd","ide"))

yslist <- list()

for(w in ys){ # w <- ys[1]

print(w)

cvr <- 20

LL <- LETTERS[1:5]

PAS <- as.data.frame(matrix(NA, cvr,5)) #cvr rounds and 5-fold

colnames(PAS) <- LL

PAS2 <- PAS3 <- PAS4 <- PAS

for(k in 1:cvr){ # for 50 rounds of 5-fold CV

print(k)

## define CV groups

groups.cv <- sample(rep(LETTERS[1:5],10000),nrow(prov.pop))

for(l in LL){ # l <- LL[1] # for each CV group

prov.pop2 <- prov.pop

rem <- which(groups.cv == l)

prov.pop2[rem,w] <- NA

fixf <- as.formula(paste(w,"~1"))

## additive model

mix.pop <- mmer2(fixf,

random = ~ g(id),

rcov=~ units,iters = 40,

G=list(id=A.pop), silent = TRUE,

data=prov.pop2)

PAS[k,l] <- cor(prov.pop[rem,w],fitted(mix.pop)[rem], use="complete")

## add+dom model

mix.pop <- mmer2(fixf,

random = ~ g(id)+g(idd),

rcov=~ units,iters = 40,

G=list(id=A.pop,idd=D.pop),silent = TRUE,

data=prov.pop2)

PAS2[k,l] <- cor(prov.pop[rem,w],fitted(mix.pop)[rem], use="complete")

## add+dom+epis model

mix.pop <- mmer2(fixf,

random = ~ g(id)+g(idd)+g(ide),

rcov=~ units,iters = 40,

G=list(id=A.pop,idd=D.pop,ide=E.pop),silent = TRUE,

data=prov.pop2)

PAS3[k,l] <- cor(prov.pop[rem,w],fitted(mix.pop)[rem], use="complete")

## mgblup model

if(u != "cnj04"){

fixf <- as.formula(paste("cbind(",paste(ys,collapse = ","),") ~1"))

mix.pop <- mmer2(fixf,

random = ~ us(trait):g(id),

rcov=~ us(trait):units,iters = 50,

G=list(id=A.pop),silent = TRUE,

data=prov.pop2)

mv <- fitted(mix.pop); colnames(mv) <- ys

PAS4[k,l] <- cor(prov.pop[rem,w],mv[rem,w], use="complete")

}

}# end of for each cv group

}# end of the k.th CV round

PAS$TRAIT <- itrait;PAS2$TRAIT <- itrait;PAS3$TRAIT <- itrait;PAS4$TRAIT <- itrait

PAS$YEAR <- w;PAS2$YEAR <- w;PAS3$YEAR <- w;PAS4$YEAR <- w

PAS$POP <- u;PAS2$POP <- u;PAS3$POP <- u;PAS4$POP <- u

PAS$method <- "add";PAS2$method <- "dom";PAS3$method <- "epi";PAS4$method <- "madd"

yslist[[w]] <- as.data.frame(rbind(PAS,PAS2,PAS3,PAS4))

}## end of for each year

ysdata <- data.frame(do.call(rbind,yslist))

popLIST[[u]] <- ysdata

}

popdata <- as.data.frame(do.call(rbind, popLIST))

traitLIST[[itrait]] <- popdata

}

# plot for each trait

# comparing methods within pops

lapply(traitLIST, function(x){

#layout(matrix(1:6,2,3))

sp1 <- x#split(x,x[,"POP"]) # split by pops

sp1$across <- apply(sp1[,LETTERS[1:5]],1,mean,na.rm=TRUE)

# qlist <- list()

# for(q in 1:5){

# qlist[[q]]<-sp1[,c(q,6:ncol(sp1))]

# colnames(qlist[[q]])[1] <- "across"

# }

# sp1 <- as.data.frame(do.call(rbind,qlist))

#

sp1$yearpop <- paste(sp1[,"YEAR"], sp1[,"POP"])

print(

# bwplot(across~method|yearpop, data=sp1, ylim=c(0,1),

# ylab="PA", xlab="Method", layout=c(3,2), col=2:5,

# main="Predictive ability"

# )

uu<- qplot(method, across, data = sp1, fill=method, ylim=c(0,1) ) +

geom_boxplot() + facet_grid(~yearpop) + scale_fill_brewer()

)

})

names(traitLIST)

library(ggplot2)

head(traitLIST$yield)

?reshape

head(traitLIST$yield)

rownames(traitLIST$yield) <- NULL

prox <- droplevels(traitLIST$yield)

prox$uni <- 1:nrow(prox)

prov00 <- reshape(prox,

idvar = c("uni","method","YEAR","POP","TRAIT"),

varying = list(1:5),

v.names = "pa", direction = "long")

head(prov00)

tab1 <- aggregate(pa~TRAIT+method+YEAR+POP, data = prov00, FUN = function(x){c(mean(x),sd(x))})

prox <- droplevels(traitLIST$wpfruit)

prox$uni <- 1:nrow(prox)

prov00 <- reshape(prox,

idvar = c("uni","method","YEAR","POP","TRAIT"),

varying = list(1:5),

v.names = "pa", direction = "long")

head(prov00)

tab2 <- aggregate(pa~TRAIT+method+YEAR+POP, data = prov00, FUN = function(x){c(mean(x),sd(x))})

tab2

tab3 <- rbind(tab1,tab2)

head(tab3)

tab3$TRAIT[which(tab3$TRAIT == "wpfruit")] <- "MFW"

tab3$TRAIT[which(tab3$TRAIT == "yield")] <- "TY"

tab3$method[which(tab3$method == "add")] <- "A"

tab3$method[which(tab3$method == "dom")] <- "AD"

tab3$method[which(tab3$method == "epi")] <- "ADE"

tab3$method[which(tab3$method == "madd")] <- "MA"

tab3$POP[which(tab3$POP == "cnj02")] <- "CNJ02"

tab3$POP[which(tab3$POP == "cnj04")] <- "CNJ04"

tab3$POP[which(tab3$POP == "grig")] <- "GRYG"

head(tab3)

tab3 <- data.frame(tab3[,1:4], tab3$pa[,1], tab3$pa[,2])

colnames(tab3)[5:6] <- c("PA.mu","PA.sd")

write.csv(tab3,file=file.path(outdir,"by_year_PAs.csv"))

###########################################

## cross validation for GS across years

## 5 fold

## MGBLUP uses data from the additional trait

###########################################

pops <- c("grig","cnj02","cnj04")

# pop

popLISTA <- list()

for(u in pops){ # u <- pops[1]

print(u)

prov.pop <- acrossblups0[[u]]

#colnames(prov.pop)[which(colnames(prov.pop) != "id")] <- paste("Y",colnames(prov.pop)[which(colnames(prov.pop) != "id")],sep="")

#if(u=="cnj02"){prov.pop <- prov.pop[,setdiff(colnames(prov.pop),"Y2013")]}

#if(u=="cnj04"){prov.pop <- prov.pop[,setdiff(colnames(prov.pop),c("Y2011","Y2014"))]}

prov.pop[which(prov.pop == 0, arr.ind = TRUE)] <- NA

A.pop <- A[[u]]

D.pop <- D[[u]]

E.pop <- E[[u]]

common.pop <- intersect(rownames(A.pop), prov.pop$id)

prov.pop <- prov.pop[which(prov.pop$id %in% common.pop),]

prov.pop$idd <- prov.pop$id

prov.pop$ide <- prov.pop$id

# year

ts <- setdiff(colnames(prov.pop), c("id","idd","ide"))

tslist <- list()

for(w in ts){ # w <- ts[1]

print(w)

cvr <- 30

LL <- LETTERS[1:5]

PAS <- as.data.frame(matrix(NA, cvr,5)) #cvr rounds and 5-fold

colnames(PAS) <- LL

PAS2 <- PAS3 <- PAS4 <- PAS

for(k in 1:cvr){ # for 50 rounds of 5-fold CV

print(k)

## define CV groups

groups.cv <- sample(rep(LETTERS[1:5],10000),nrow(prov.pop))

for(l in LL){ # l <- LL[1] # for each CV group

prov.pop2 <- prov.pop

rem <- which(groups.cv == l)

prov.pop2[rem,w] <- NA

fixf <- as.formula(paste(w,"~1"))

## additive model

mix.pop <- mmer2(fixf,

random = ~ g(id),

rcov=~ units,iters = 40,

G=list(id=A.pop), silent = TRUE,

data=prov.pop2)

PAS[k,l] <- cor(prov.pop[rem,w],fitted(mix.pop)[rem], use="complete")

## add+dom model

mix.pop <- mmer2(fixf,

random = ~ g(id)+g(idd),

rcov=~ units,iters = 40,

G=list(id=A.pop,idd=D.pop),silent = TRUE,

data=prov.pop2)

PAS2[k,l] <- cor(prov.pop[rem,w],fitted(mix.pop)[rem], use="complete")

## add+dom+epis model

mix.pop <- mmer2(fixf,

random = ~ g(id)+g(idd)+g(ide),

rcov=~ units,iters = 40,

G=list(id=A.pop,idd=D.pop,ide=E.pop),silent = TRUE,

data=prov.pop2)

PAS3[k,l] <- cor(prov.pop[rem,w],fitted(mix.pop)[rem], use="complete")

## mgblup model

fixf <- as.formula(paste("cbind(",paste(ts,collapse = ","),") ~1"))

mix.pop <- mmer2(fixf,

random = ~ us(trait):g(id),

rcov=~ us(trait):units,iters = 50,

G=list(id=A.pop),silent = TRUE,

data=prov.pop2)

mv <- fitted(mix.pop); colnames(mv) <- ts

PAS4[k,l] <- cor(prov.pop[rem,w],mv[rem,w], use="complete")

}# end of for each cv group

}# end of the k.th CV round

PAS$TRAIT <- w;PAS2$TRAIT <- w;PAS3$TRAIT <- w;PAS4$TRAIT <- w

#PAS$YEAR <- w;PAS2$YEAR <- w;PAS3$YEAR <- w;PAS4$YEAR <- w

PAS$POP <- u;PAS2$POP <- u;PAS3$POP <- u;PAS4$POP <- u

PAS$method <- "add";PAS2$method <- "dom";PAS3$method <- "epi";PAS4$method <- "madd"

tslist[[w]] <- as.data.frame(rbind(PAS,PAS2,PAS3,PAS4))

}## end of for each year

tsdata <- data.frame(do.call(rbind,tslist))

popLISTA[[u]] <- tsdata

}

popdata <- as.data.frame(do.call(rbind, popLISTA))

head(popdata)

# plot for each trait

# comparing methods within pops

library(ggplot2)

sp1 <- popdata#split(x,x[,"POP"]) # split by pops

sp1$across <- apply(sp1[,LETTERS[1:5]],1,mean,na.rm=TRUE)

sp1$TRAIT[which(sp1$TRAIT == "wpfruit")] <- "MFW"

sp1$TRAIT[which(sp1$TRAIT == "yield")] <- "TY"

sp1$method[which(sp1$method == "add")] <- "A"

sp1$method[which(sp1$method == "dom")] <- "AD"

sp1$method[which(sp1$method == "epi")] <- "ADE"

sp1$method[which(sp1$method == "madd")] <- "MA"

sp1$POP[which(sp1$POP == "cnj02")] <- "CNJ02"

sp1$POP[which(sp1$POP == "cnj04")] <- "CNJ04"

sp1$POP[which(sp1$POP == "grig")] <- "GRYG"

sp1$yearpop <- paste(sp1[,"TRAIT"], sp1[,"POP"])

colnames(sp1)[which(colnames(sp1) == "method")] <- "Method"

colnames(sp1)[which(colnames(sp1) == "across")] <- "PA"

print(

uu<- qplot(Method, PA, data = sp1, fill=Method, ylim=c(0,1) ) +

geom_boxplot() + facet_grid(~yearpop)

#+ scale_fill_brewer()

+ scale_fill_manual(values = rep("cadetblue",4), labels = c("A: GBLUP-A","AD: GBLUP-AD","ADE: GBLUP-ADE","MA: MGBLUP-A"))

)

head(sp1)

prox <- sp1[,-which(colnames(sp1)=="PA")]

head(prox)

prox$uni <- 1:nrow(prox)

prov000 <- reshape(prox,

idvar = c("uni","Method","POP","TRAIT"),

varying = list(1:5),

v.names = "PA", direction = "long")

head(prov000,10)

tab4 <- aggregate(PA~Method+POP+TRAIT, data = prov000, FUN = mean)

tab4

write.csv(tab4,file=file.path(outdir,"by_trait_PAs.csv"))

# load("~/Desktop/VMGSIC/new version 2018/pheno and geno/GSpaper_02_results.RData")

xx <- as.data.frame(do.call(rbind, traitLIST))

head(xx)

table(xx$TRAIT)

prox <- xx

prox$uni <- 1:nrow(prox)

prox$across <- apply(prox[,LETTERS[1:5]],1,mean,na.rm=TRUE)

head(prox)

prox$TRAIT[which(prox$TRAIT == "wpfruit")] <- "MFW"

prox$TRAIT[which(prox$TRAIT == "yield")] <- "TY"

prox$method[which(prox$method == "add")] <- "A"

prox$method[which(prox$method == "dom")] <- "AD"

prox$method[which(prox$method == "epi")] <- "ADE"

prox$method[which(prox$method == "madd")] <- "MA"

prox$POP[which(prox$POP == "cnj02")] <- "CNJ02"

prox$POP[which(prox$POP == "cnj04")] <- "CNJ04"

prox$POP[which(prox$POP == "grig")] <- "GRYG"

prox$typ <- paste(prox$TRAIT, prox$POP, prox$YEAR)

colnames(prox)[which(colnames(prox) == "method")] <- "Method"

colnames(prox)[which(colnames(prox) == "across")] <- "PA"

print(

uu<- qplot(Method, PA, data = prox, fill=Method, ylim=c(0,1) ) +

geom_boxplot()

#+ facet_grid(~typ)

#+ scale_fill_brewer()

+ facet_wrap(~typ, nrow = 2)

+ scale_fill_manual(values = rep("cadetblue",4), labels = c("A: GBLUP-A","AD: GBLUP-AD","ADE: GBLUP-ADE","MA: MGBLUP-A"))

# + scale_x_discrete(labels=as.character(unique(prox$Method)),

# breaks=c("GBLUP-A","GBLUP-AD","GBLUP-ADE","MGBLUP-A"))

)

#922 x 535
