# Supplementary material for: Multivariate GBLUP Improves Accuracy of Genomic Selection for Yield and Fruit Weight in Biparental Populations of Vaccinium macrocarpon Ait
Source: Front Plant Sci. 2018 Sep 12;9:1310. doi: 10.3389/fpls.2018.01310 (PMC6144488; doi:10.3389/fpls.2018.01310)
Supplement: Supplementary File 4 — Effect of marker density in PA. [file Table_4.docx]

**Supplementary File 4**

###########################################

## EFFECT OF MARKER DENSITY IN PREDICTION

# using across year estimates

###########################################

rm(list=ls())

load("~/Desktop/VMGSIC/new version 2018/pheno and geno/GSpaper_01_results.RData")

map <- openxlsx::read.xlsx("POPS3_ALL.xlsx" ,sheet=8)

head(map)

map <- map[which(map$LG %in% 1:12),]

#map$Position <- round(map$Position)

map$total <- map$Position

prox <-0

# make total map distance

for(o in 1:12){

if(o == 1){

map$total[which(map$LG == o)] <- map$total[which(map$LG == o)]

}else{

map$total[which(map$LG == o)] <- map$total[which(map$LG == o)] + prox

}

prox <- prox + max(map$Position[which(map$LG == o)])

}

head(map)

plot(map$total) # ~1200 cM

## create the number of bins required to have this amount of markers picked

## create 20, 50, ... bins, so nmar=nbins since we are going to sample one marker per bin

nmar <- c(20, 50, 100, 250, 500, 750, 1000) # the total map only span over 1285 cM

# make new columns and divisions in bins

foonames <- character()

for(o in 1:length(nmar)){ # for each number of bins=nmar to sample

foo <- round(max(map$total)/nmar[o],3) # foo cM should cover each bin

foonames[o] <- paste("cM",foo, sep=".")

map[,paste("cM",foo, sep=".")] <- NA

st <- seq(0,max(map$total),foo)

#st <- seq(1,nrow(map),foo)

en <- c((st)[-1],max(map$total))

for(k in 1:length(st)){

map[which(map$total >= st[k] & map$total <= en[k]),paste("cM",foo, sep=".")] <- k

#map[st[k]:en[k],paste("nm",nmar[o], sep="_")] <- k

}

}

head(map)

foonames # bins split at every x cM

nmar # to sample this number of markers,

unique(map$cM.1.202)

# i.e if we have one bin every cM we have 20 bins and we will sample one marker per bin

# assuming they are all close together

Mlist <- list(grig=M.grig, cnj02=M.cnj02, cnj04=M.cnj04)

traitPAlist <- list()

for(itrait in traits){ # itrait <- traits[1] # for each trait

print(itrait)

#data0 <- yearblups[[itrait]]

pops <- c("grig","cnj02","cnj04")

# pop

popLIST <- list()

PASlist <- list()

for(u in pops){ # u <- pops[1] # for each pop

print(u)

Mprov <- Mlist[[u]]# marker data

prov.data <- acrossblups0[[u]] # get population blups

keepid <- intersect(prov.data$id, rownames(Mprov))

prov.data <- prov.data[which(prov.data$id %in% keepid),]

prov.data2 <- prov.data

vp <- sample(1:nrow(prov.data), round(nrow(prov.data)/5))

prov.data2[vp,itrait] <- NA

iters=1000

PAS <- matrix(NA, iters, length(nmar))

for(iit in 1:iters){ # iit <- 1

print(paste(iit,"iteration"))

for(ee in 1:length(nmar)){ # ee <- 1 # for each number of markers

print(paste(ee,"density"))

#head(map[which(is.na(map$cM.60)),])

bins <- unique(map[,foonames[ee]])

map.prov <- map[which(map$Locus %in% colnames(Mprov)),]

vv <- map.prov[,foonames[ee]]

take <- apply(data.frame(bins),1,function(x){

found <- which(vv == x)

if(length(found)>0){return(sample(found,1))}else{return(NA)}

})

take <- na.omit(take)

markers.taken <- map.prov$Locus[take]

Ap <- A.mat(Mprov[,markers.taken])

## make the modeling

## additive model

fixf <- as.formula(paste(itrait,"~1"))

mix.pop <- mmer2(fixf,

random = ~ g(id),

rcov=~ units,iters = 40,

G=list(id=Ap), silent = TRUE,

data=prov.data2)

PAS[iit,ee] <- cor(prov.data[vp,itrait],fitted(mix.pop)[vp], use="complete")

}

}

#head(PAS)

PASlist[[u]] <- PAS

}

traitPAlist[[itrait]] <- PASlist

}

outdir <- "~/Desktop/VMGSIC/new version 2018/pheno and geno"

save.image(file.path(outdir,"GSpaper_03_results.RData"))

#load(file.path(outdir,"GSpaper_03_results.RData"))

#####################

######################

## plot for PA based on density

nmar

foonames2 <- paste("1 marker every ",gsub("cM.","",foonames)," cM (", nmar,")", sep="")

mdr0 <- as.data.frame(traitPAlist$yield$grig)

mdr0$uni <- 1:nrow(mdr0)

prov0000 <- reshape(mdr0,

idvar = c("uni"),

varying = list(1:7),

v.names = "PA", direction = "long")

prov0000$time <- foonames2[prov0000$time]

prov0000$time <- factor(prov0000$time, levels = unique(prov0000$time))

prov0000$TRAIT <- "TY"

head(prov0000,10)

mdr0 <- as.data.frame(traitPAlist$wpfruit$grig)

mdr0$uni <- 1:nrow(mdr0)

prov0001 <- reshape(mdr0,

idvar = c("uni"),

varying = list(1:7),

v.names = "PA", direction = "long")

prov0001$time <- foonames2[prov0001$time]

prov0001$time <- factor(prov0001$time, levels = unique(prov0001$time))

prov0001$TRAIT <- "MFW"

head(prov0001,10)

prov0002 <- as.data.frame(rbind(prov0000, prov0001))

colnames(prov0002)[which(colnames(prov0002) == "time")] <- "Density_scenario"

print(

uu<- qplot(Density_scenario, PA, data = prov0002, fill=Density_scenario, ylim=c(0,1) ) +

geom_boxplot() + facet_grid(~TRAIT) + scale_fill_brewer()

+ theme(axis.text.x =element_text(angle = 45, hjust = 1),legend.text=element_text(size=9)) #

+ scale_x_discrete(breaks=as.character(unique(prov0002$Density_scenario)),

labels=paste("density:",nmar))

#+ guides(fill=FALSE)

)

tab5 <- aggregate(PA~time+TRAIT, data = prov0002, FUN = mean)

tab5

write.csv(tab5,file=file.path(outdir,"by_marker_density_PAs.csv"))

boxplot(abs(traitPAlist$yield$grig), ylim=c(0,0.8))

boxplot((traitPAlist$yield$cnj02), ylim=c(0,0.8))

boxplot((traitPAlist$yield$cnj04), ylim=c(0,0.8))

boxplot(abs(traitPAlist$wpfruit$grig), ylim=c(0,0.8))

boxplot(abs(traitPAlist$wpfruit$cnj02), ylim=c(0,0.8))

boxplot(abs(traitPAlist$wpfruit$cnj04), ylim=c(0,0.8))
