# Supplementary material for: Multivariate GBLUP Improves Accuracy of Genomic Selection for Yield and Fruit Weight in Biparental Populations of Vaccinium macrocarpon Ait
Source: Front Plant Sci. 2018 Sep 12;9:1310. doi: 10.3389/fpls.2018.01310 (PMC6144488; doi:10.3389/fpls.2018.01310)
Supplement: Supplementary File 5 — Cross-validation with across years BLUPs. [file Table_5.docx]

**Supplementary File 5**

rm(list=ls())

# "~/Desktop/VMGSIC/new version 2018/pheno and geno"

load("~/Desktop/VMGSIC/new version 2018/pheno and geno/GSpaper_02_results.RData")

library(sommer)

bb <- list(colnames(M.cnj02),colnames(M.cnj04),colnames(M.grig))

commonM <- Reduce(intersect,bb)

M <- rbind(M.grig[,commonM], M.cnj02[,commonM],M.cnj04[,commonM])

###########################################

## cross validation for GS across years

## 5 fold

## MGBLUP uses data from the additional trait

###########################################

pops <- c("grig","cnj02","cnj04")

# pop

popLISTA <- list()

#for(u in pops){ # u <- pops[1]

print(u)

prov.pop1 <- acrossblups0$grig

prov.pop1[which(prov.pop1 == 0, arr.ind = TRUE)] <- NA

A.pop1 <- A$grig

common.pop1 <- intersect(rownames(A.pop1), prov.pop1$id)

prov.pop1 <- prov.pop1[which(prov.pop1$id %in% common.pop1),]

prov.pop2 <- acrossblups0$cnj02

prov.pop2[which(prov.pop2 == 0, arr.ind = TRUE)] <- NA

A.pop2 <- A$cnj02

common.pop2 <- intersect(rownames(A.pop2), prov.pop2$id)

prov.pop2 <- prov.pop2[which(prov.pop2$id %in% common.pop2),]

prov.pop3 <- acrossblups0$cnj04

prov.pop3[which(prov.pop3 == 0, arr.ind = TRUE)] <- NA

A.pop3 <- A$cnj04

common.pop3 <- intersect(rownames(A.pop3), prov.pop3$id)

prov.pop3 <- prov.pop3[which(prov.pop3$id %in% common.pop3),]

# traits

(ts <- setdiff(colnames(prov.pop), c("id","idd","ide")))

tslist <- list()

for(w in ts[2]){ # w <- ts[1]

print(w)

iters=400

tpsizes <- seq(20,180,20)

PAS <- as.data.frame(matrix(NA, iters,length(tpsizes))) #cvr rounds and 5-fold

colnames(PAS) <- paste0("tps",tpsizes)

# for each scenario TP-CNJ02.VP-CNJ02, TP-CNJ02.VP-CNJ04

PAS2 <- PAS3 <- PAS4 <- PAS5 <- PAS6 <- PAS7 <- PAS8 <- PAS9 <- PAS

# PAS TP-GRYG.VP-GRYG

# PAS TP-CNJ02.VP-GRYG

# PAS TP-CNJ04.VP-GRYG

# PAS TP-GRYG.VP-CNJ02

# PAS TP-CNJ02.VP-CNJ02

# PAS TP-CNJ04.VP-CNJ02

# PAS TP-GRYG.VP-CNJ04

# PAS TP-CNJ02.VP-CNJ04

# PAS TP-CNJ04.VP-CNJ04

for(i in 1:iters){

print(paste("iter",i))

for(k in 1:length(tpsizes)){ # for 50 rounds of 5-fold CV

print(k)

## define CV groups

tps <- tpsizes[k]

vps <- 20

###########################

###########################

### scenario 1

# PAS TP-CNJ02.VP-GRYG

vp <- prov.pop1[sample(1:nrow(prov.pop1),vps),]

vp2 <- vp; vp2[,w] <- NA

if(tps <= nrow(prov.pop1)){

tp <- prov.pop1[sample(1:nrow(prov.pop1),tps),]

# phenotype

dos1 <- as.data.frame(rbind(vp,tp))

dos2 <- as.data.frame(rbind(vp2,tp))

# genotypes

Mdos <- M[dos1$id,]

Ados <- A.mat(Mdos)

fixf <- as.formula(paste(w,"~1"))

## additive model

mix.pop <- mmer2(fixf,

random = ~ g(id),

rcov=~ units,iters = 40,

G=list(id=Ados), silent = TRUE,

data=dos2)

PAS[i,k] <- cor(dos1[1:vps,w],fitted(mix.pop)[1:vps], use="complete")

}

###########################

###########################

### scenario 2

# PAS TP-CNJ04.VP-GRYG

vp <- prov.pop1[sample(1:nrow(prov.pop1),vps),]

vp2 <- vp; vp2[,w] <- NA

if(tps <= nrow(prov.pop2)){

tp <- prov.pop2[sample(1:nrow(prov.pop2),tps),]

# phenotype

dos1 <- as.data.frame(rbind(vp,tp))

dos2 <- as.data.frame(rbind(vp2,tp))

# genotypes

Mdos <- M[dos1$id,]

Ados <- A.mat(Mdos)

fixf <- as.formula(paste(w,"~1"))

## additive model

mix.pop <- mmer2(fixf,

random = ~ g(id),

rcov=~ units,iters = 40,

G=list(id=Ados), silent = TRUE,

data=dos2)

PAS2[i,k] <- cor(dos1[1:vps,w],fitted(mix.pop)[1:vps], use="complete")

}

###########################

###########################

### scenario 3

# PAS TP-CNJ04.VP-GRYG

vp <- prov.pop1[sample(1:nrow(prov.pop1),vps),]

vp2 <- vp; vp2[,w] <- NA

if(tps <= nrow(prov.pop3)){

tp <- prov.pop3[sample(1:nrow(prov.pop3),tps),]

# phenotype

dos1 <- as.data.frame(rbind(vp,tp))

dos2 <- as.data.frame(rbind(vp2,tp))

# genotypes

Mdos <- M[dos1$id,]

Ados <- A.mat(Mdos)

fixf <- as.formula(paste(w,"~1"))

## additive model

mix.pop <- mmer2(fixf,

random = ~ g(id),

rcov=~ units,iters = 40,

G=list(id=Ados), silent = TRUE,

data=dos2)

PAS3[i,k] <- cor(dos1[1:vps,w],fitted(mix.pop)[1:vps], use="complete")

}

###########################

###########################

### scenario 4

# PAS TP-GRYG.VP-CNJ02

vp <- prov.pop2[sample(1:nrow(prov.pop2),vps),]

vp2 <- vp; vp2[,w] <- NA

if(tps <= nrow(prov.pop1)){

tp <- prov.pop1[sample(1:nrow(prov.pop1),tps),]

# phenotype

dos1 <- as.data.frame(rbind(vp,tp))

dos2 <- as.data.frame(rbind(vp2,tp))

# genotypes

Mdos <- M[dos1$id,]

Ados <- A.mat(Mdos)

fixf <- as.formula(paste(w,"~1"))

## additive model

mix.pop <- mmer2(fixf,

random = ~ g(id),

rcov=~ units,iters = 40,

G=list(id=Ados), silent = TRUE,

data=dos2)

PAS4[i,k] <- cor(dos1[1:vps,w],fitted(mix.pop)[1:vps], use="complete")

}

###########################

###########################

### scenario 5

# PAS TP-CNJ02.VP-CNJ02

vp <- prov.pop2[sample(1:nrow(prov.pop2),vps),]

vp2 <- vp; vp2[,w] <- NA

if(tps <= nrow(prov.pop2)){

tp <- prov.pop2[sample(1:nrow(prov.pop2),tps),]

# phenotype

dos1 <- as.data.frame(rbind(vp,tp))

dos2 <- as.data.frame(rbind(vp2,tp))

# genotypes

Mdos <- M[dos1$id,]

Ados <- A.mat(Mdos)

fixf <- as.formula(paste(w,"~1"))

## additive model

mix.pop <- mmer2(fixf,

random = ~ g(id),

rcov=~ units,iters = 40,

G=list(id=Ados), silent = TRUE,

data=dos2)

PAS5[i,k] <- cor(dos1[1:vps,w],fitted(mix.pop)[1:vps], use="complete")

}

###########################

###########################

### scenario 6

# PAS TP-CNJ04.VP-CNJ02

vp <- prov.pop2[sample(1:nrow(prov.pop2),vps),]

vp2 <- vp; vp2[,w] <- NA

if(tps <= nrow(prov.pop3)){

tp <- prov.pop3[sample(1:nrow(prov.pop3),tps),]

# phenotype

dos1 <- as.data.frame(rbind(vp,tp))

dos2 <- as.data.frame(rbind(vp2,tp))

# genotypes

Mdos <- M[dos1$id,]

Ados <- A.mat(Mdos)

fixf <- as.formula(paste(w,"~1"))

## additive model

mix.pop <- mmer2(fixf,

random = ~ g(id),

rcov=~ units,iters = 40,

G=list(id=Ados), silent = TRUE,

data=dos2)

PAS6[i,k] <- cor(dos1[1:vps,w],fitted(mix.pop)[1:vps], use="complete")

}

###########################

###########################

### scenario 7

# PAS TP-GRYG.VP-CNJ04

vp <- prov.pop3[sample(1:nrow(prov.pop3),vps),]

vp2 <- vp; vp2[,w] <- NA

if(tps <= nrow(prov.pop1)){

tp <- prov.pop1[sample(1:nrow(prov.pop1),tps),]

# phenotype

dos1 <- as.data.frame(rbind(vp,tp))

dos2 <- as.data.frame(rbind(vp2,tp))

# genotypes

Mdos <- M[dos1$id,]

Ados <- A.mat(Mdos)

fixf <- as.formula(paste(w,"~1"))

## additive model

mix.pop <- mmer2(fixf,

random = ~ g(id),

rcov=~ units,iters = 40,

G=list(id=Ados), silent = TRUE,

data=dos2)

PAS7[i,k] <- cor(dos1[1:vps,w],fitted(mix.pop)[1:vps], use="complete")

}

###########################

###########################

### scenario 8

# PAS TP-CNJ02.VP-CNJ04

vp <- prov.pop3[sample(1:nrow(prov.pop3),vps),]

vp2 <- vp; vp2[,w] <- NA

if(tps <= nrow(prov.pop2)){

tp <- prov.pop2[sample(1:nrow(prov.pop2),tps),]

# phenotype

dos1 <- as.data.frame(rbind(vp,tp))

dos2 <- as.data.frame(rbind(vp2,tp))

# genotypes

Mdos <- M[dos1$id,]

Ados <- A.mat(Mdos)

fixf <- as.formula(paste(w,"~1"))

## additive model

mix.pop <- mmer2(fixf,

random = ~ g(id),

rcov=~ units,iters = 40,

G=list(id=Ados), silent = TRUE,

data=dos2)

PAS8[i,k] <- cor(dos1[1:vps,w],fitted(mix.pop)[1:vps], use="complete")

}

###########################

###########################

### scenario 9

# PAS TP-CNJ04.VP-CNJ04

vp <- prov.pop3[sample(1:nrow(prov.pop3),vps),]

vp2 <- vp; vp2[,w] <- NA

if(tps <= nrow(prov.pop3)){

tp <- prov.pop3[sample(1:nrow(prov.pop3),tps),]

# phenotype

dos1 <- as.data.frame(rbind(vp,tp))

dos2 <- as.data.frame(rbind(vp2,tp))

# genotypes

Mdos <- M[dos1$id,]

Ados <- A.mat(Mdos)

fixf <- as.formula(paste(w,"~1"))

## additive model

mix.pop <- mmer2(fixf,

random = ~ g(id),

rcov=~ units,iters = 40,

G=list(id=Ados), silent = TRUE,

data=dos2)

PAS9[i,k] <- cor(dos1[1:vps,w],fitted(mix.pop)[1:vps], use="complete")

}

}# end of the k.th CV round

}

PROV <- list(PAS,PAS2,PAS3,PAS4,PAS5,PAS6,PAS7,PAS8,PAS9)

PROV <- lapply(PROV,function(x){x$TRAIT <- w; x$Method <- "A"; return(x)})

PROV <- lapply(PROV, as.data.frame)

PROV <- lapply(PROV, function(x){x$uni <- 1:nrow(x); return(x)})

sss <- c("TP-GRYG.VP-GRYG","TP-CNJ02.VP-GRYG","TP-CNJ04.VP-GRYG",

"TP-GRYG.VP-CNJ02","TP-CNJ02.VP-CNJ02","TP-CNJ04.VP-CNJ02",

"TP-GRYG.VP-CNJ04","TP-CNJ02.VP-CNJ04","TP-CNJ04.VP-CNJ04")

for(i in 1:length(PROV)){

PROV[[i]]$Scenario <- sss[i]

}

PROV2 <-lapply(PROV,function(x){

prov000 <- reshape(x[,c(1:9,12)],

idvar = c("uni"),

varying = list(1:9),

v.names = "PA", direction = "long")

prov000$TRAIT <- w

return(prov000)

})

for(i in 1:length(PROV2)){

PROV2[[i]]$Scenario <- sss[i]

}

#PROV$uni <- 1:nrow(PROV)

tslist[[w]] <- PROV2

}## end of for each year

#}

names(tslist)

apply(PAS,2,mean, na.rm=TRUE)

boxplot(PAS9)

# plot for each trait

# comparing methods within pops

step1 <- lapply(tslist,function(x){

as.data.frame(do.call(rbind,x))

}

)

step2 <- as.data.frame(do.call(rbind, step1))

step2$TRAIT[which(step2$TRAIT == "wpfruit")] <- "MFW"

step2$TRAIT[which(step2$TRAIT == "yield")] <- "TY"

head(step2)

step2$time <- paste("TP",tpsizes,sep="-")[step2$time]

step2$time <- factor(step2$time, paste("TP",tpsizes,sep="-"))

head(step2)

step2$ts <- paste(step2$TRAIT, levels=step2$Scenario)

head(step2)

library(ggplot2)

colnames(step2)[which(colnames(step2) == "time")] <- "Scenario"

pp1 <- ggplot(step2[which(step2$TRAIT=="MFW"),], aes(Scenario, PA, fill=Scenario)) +

facet_wrap(~ts, nrow = 3) + #facet_grid(. ~ ts) +

geom_boxplot(aes(group = Scenario), outlier.alpha = 0.1) +

theme(axis.text.x = element_text(angle = 45, hjust = 1)) +

scale_fill_brewer() + theme(legend.position="none")

#+ facet_wrap(~ts, nrow = 3)

pp2 <- ggplot(step2[which(step2$TRAIT=="TY"),], aes(Scenario, PA, fill=Scenario)) +

facet_wrap(~ts, nrow = 3) + #facet_grid(. ~ ts) +

geom_boxplot(aes(group = Scenario), outlier.alpha = 0.1) +

theme(axis.text.x = element_text(angle = 45, hjust = 1)) +

scale_fill_brewer() + theme(legend.position="none")

library(gridExtra)

grid.arrange(pp1, pp2, nrow = 1)

#

# step2$add <- paste(step2$Scenario, step2$ts)

#

# ggplot(data=step2, aes(x=time, y=PA, color = Scenario)) +

# facet_wrap(~ts, nrow = 3) +

# geom_point() +

# theme(legend.position="right") +

# labs(title="") +

# geom_smooth(method = "loess", alpha = .1)

#

# ggplot(data=Births78, aes(x=dayofyear, y=births, color = day_of_week)) +

# geom_point() +

# theme(legend.position="right") +

# labs(title="") +

# geom_smooth(method = "loess", alpha = .1)

# head(step2)

# step3 <- aggregate(PA~time+Scenario, data=step2, FUN = mean)

# step3

# ggplot(data = step2, aes(x=time, y=PA, color = Scenario)) +

# geom_line()

#
